# Metric Quality of Intelligence Tests in Spain: Evidence from 13 National Test Commission Reviews

**DOI:** 10.3390/jintelligence14090224

**Published:** 2026-09-18

**Authors:** Sergio Escorial

**Affiliations:** Department of Psychobiology and Methodology in Behavioral Sciences, Faculty of Psychology, Universidad Complutense de Madrid, Campus de Somosaguas, Pozuelo de Alarcón, 28223 Madrid, Spain; sergio.escorial@psi.ucm.es

**Keywords:** intelligence tests, psychometric quality, test review, test manuals, CET-R

## Abstract

Intelligence test scores underpin high-stakes educational, clinical, and occupational decisions, which makes the metric quality of these instruments a central concern for professional practice. This study aimed to evaluate the psychometric and documentary quality of intelligence tests reviewed by the Spanish National Test Commission, compare this quality with that of tests measuring other psychological constructs, and examine the influence of publisher and evaluation cohort on quality. To this end, 37 intelligence tests and 78 tests of other constructs, reviewed across 13 editions (2010–2026) of the project, were coded using the Revised Test Review Questionnaire (CET-R), which comprises 14 quality characteristics grouped into four dimensions: General, Validity, Reliability, and Norms. Intelligence tests did not report more information than tests of other constructs, showing similar omission rates. However, when information was reported, intelligence tests obtained higher scores on the General, Validity, and Norms dimensions, with no differences in Reliability. Publisher accounted for a substantial share of the variance in quality, whereas the evaluation cohort showed no significant influence on any psychometric characteristic once corrected for multiple comparisons (FDR). Item bias analyses and item-response-theory-based reliability remained the most consistently underreported forms of evidence across the manuals of all tests analyzed. These findings underscore the need to uphold high, transparent standards in the documentation of intelligence tests and provide empirical criteria to support the responsible selection of instruments by practitioners.

## 1. Introduction

Intelligence test scores underpin educational, clinical, neuropsychological, and occupational decisions with a notable impact on the lives of the people evaluated (Haier et al., 2023). The value of a score does not lie only in its apparent usefulness. It also lies in the strength of the inferences it supports, in the technical quality of the instrument, and in the appropriateness of its use for a specific purpose (AERA et al., 2014). Measuring intelligence rigorously is not only a methodological requirement. It is also an ethical commitment, because an inadequate interpretation can lead to unfair, biased, or clinically incorrect decisions (Escorial et al., 2006; Hernández et al., 2022). This ethical commitment also demands transparency: practitioners who administer a test need to be able to verify, in its manual, the evidence on which its scores rest.

This study adopts a definition of intelligence aligned with the classical psychometric tradition and its current developments. From this perspective, intelligence is a general cognitive capacity to reason, solve problems, learn from experience, and process information efficiently. This capacity manifests itself in an organized set of more specific abilities (Haier et al., 2023; Schneider & McGrew, 2012). Carroll (1993) synthesized this tradition in *Human Cognitive Abilities*. His work proposes a hierarchical taxonomy based on the reanalysis of more than 400 empirical datasets (Carroll, 1993; McGrew, 2023).

Carroll’s three-stratum model organizes intelligence into three levels. A top level captures a general ability, or g. A middle level groups broad abilities, such as fluid intelligence, crystallized intelligence, memory, and processing speed. A bottom level captures specific, or narrow, abilities (Carroll, 1993; McGrew, 2023). This model continues to reinforce its central role in current psychometric research. It integrates accumulated factor-analytic findings and serves as a foundation for broader, applied models (McGill & Dombrowski, 2019; McGrew, 2023).

Building on this foundation, Cattell–Horn–Carroll (CHC) theory emerged. This theory integrates Cattell and Horn’s fluid and crystallized intelligence with Carroll’s three-stratum model (McGill & Dombrowski, 2019; Schneider & McGrew, 2012). It now serves as the standard reference framework, both for developing intelligence batteries and for interpreting their scores in clinical and educational settings. Recent studies confirm that the CHC model allows meaningful comparisons across different batteries (Caemmerer et al., 2020). The specialized literature further notes that this framework has become a common language among test developers, users, and reviewers (McGill & Dombrowski, 2019; McGrew, 2023).

Many of the intelligence tests, including the Wechsler scales and various aptitude batteries, were designed according to hierarchical models of cognitive ability. This allows a direct correspondence with the CHC framework (Guilera & Barrios, 2025; Hernández et al., 2015; Muñiz et al., 2011). This study therefore interprets these tests’ quality indicators by examining whether the evidence in their manuals is consistent with the demands of hierarchical intelligence models, particularly regarding theoretical foundation, internal structure, and validity evidence (Escorial, 2026; Hernández et al., 2016).

Individual differences in cognitive ability relate to many educational, occupational, and adaptive outcomes. These relationships, however, require cautious interpretation within well-defined theoretical frameworks (Haier et al., 2023). Intelligence tests are therefore instruments of great applied relevance. But their responsible use requires solid evidence of validity, reliability, norms, and interpretability, together with clear and up-to-date technical documentation (AERA et al., 2014; Evers et al., 2013). The question is not only whether it is worth measuring intelligence. The question is how to do so in a scientifically grounded and socially responsible way (Haier et al., 2023; Hunt, 2011). When a manual fails to document this evidence clearly, practitioners are left without objective criteria to decide whether that test is appropriate for their specific case. To help address this gap, the present study pursues three specific objectives, examined in detail below: analyzing the psychometric and technical quality of intelligence tests, comparing this quality with that of instruments measuring other psychological constructs, and examining the influence of publisher and evaluation cohort on that quality.

This concern for measurement quality has driven the creation of different test-review systems. The *Standards for Educational and Psychological Testing* constitute the most influential international reference. They evaluate the quality of the inferences drawn from scores and the responsibility of the professionals who apply them (AERA et al., 2014). In Europe, the European Federation of Psychologists’ Associations (EFPA) developed its own model to describe, review, and evaluate psychological and educational tests. This model provides users with rigorous, independent information on the theoretical, practical, and psychometric characteristics of each test (Evers et al., 2013).

The EFPA model has been progressively updated to incorporate recent developments in measurement (Hernández et al., 2022). Its underlying philosophy is especially relevant for intelligence tests, because it does not merely assess isolated psychometric indicators. It proposes a comprehensive review that includes the quality of materials, theoretical foundation, adaptation, validity, reliability, norms, and the level of detail of the available documentation (Evers et al., 2013). The quality of a test is thus no longer understood as a single property. It comes to be understood as the result of several sources of evidence and different criteria of appropriate use (AERA et al., 2014).

In Spain, Prieto and Muñiz (2000) adapted these early European developments and proposed their own model for evaluating test quality. This model was embodied in the Test Evaluation Questionnaire (Cuestionario para la Evaluación de los Tests [CET]), designed to combine descriptive information, quantitative rating, and qualitative judgment (Ponsoda & Hontangas, 2013; Prieto & Muñiz, 2000). The subsequent evolution of psychometrics prompted a revision of the instrument. This gave rise to the Revised Test Evaluation Questionnaire (CET-R), aligned with the revised European model and incorporating advances in validity, reliability, adaptation, and norms (Fonseca-Pedrero & Muñiz, 2017; Hernández et al., 2016). More recently, the CET-R v1.1 was developed. This version introduces more precise criteria for distinguishing between missing information and non-applicable aspects, along with guidance for adapted tests (Escorial, 2026; Guilera & Barrios, 2025; Recio-Saboya, 2026).

Building on this methodological foundation, the National Test Commission (Comisión Nacional de Test [CNT]) of the Spanish General Council of Psychology (*Consejo General de la Psicología de España*) has led the *Evaluation of Tests Published in Spain* project since 2010. This project implements a systematic, independent review procedure, with the aim of providing practitioners with verified technical information (Fonseca-Pedrero & Muñiz, 2017; Guilera & Barrios, 2025; Hernández et al., 2015). The procedure has remained stable across thirteen editions: test selection, appointment of reviewers, application of the questionnaire, integration of reports, publisher appeals, and open publication of the final report (Abad, 2024; Hernández et al., 2015; Recio-Saboya, 2026). These thirteen evaluations constitute one of the most systematic efforts of public test review in the Ibero-American context (Abad, 2024; Elosua & Geisinger, 2016; Escorial, 2026; Fonseca-Pedrero & Muñiz, 2017; Gómez-Sánchez, 2019; Guilera & Barrios, 2025; Hernández et al., 2015; Hidalgo & Hernández, 2019; Lozano, 2023; Muñiz et al., 2011; Ponsoda & Hontangas, 2013; Recio-Saboya, 2026; Viladrich et al., 2021).

The project’s own historical development suggests that the review system has fostered a culture of greater technical transparency. Already in early evaluations, it was observed that some recent manuals organized their information in a way that more closely matched the CET’s sections (Hernández et al., 2015). Consistent with this, more recent analyses indicate that tests developed in later years more frequently report information on aspects such as differential item functioning or temporal stability. However, this does not always translate into statistically significant improvements in quality scores (Guilera & Barrios, 2025).

Studies on attitudes toward and use of tests in Spain show a generally positive view of these instruments among psychologists. They also show a persistent demand for more independent technical information (Muñiz et al., 2020; Muñiz & Fernández-Hermida, 2000, 2010). CNT reports serve a double function here. They guide practitioners and, at the same time, generate a cumulative repository that allows researchers to study test quality by construct, publisher, age, or evaluation cohort (Escorial, 2026; Guilera & Barrios, 2025). All reports are publicly available through the Spanish General Council of Psychology (Comisión Nacional de Test, 2026).

Despite the richness of this material, an analysis focused exclusively on intelligence tests is warranted. This focus is justified for three reasons. First, these are instruments with a long tradition, wide use, and considerable diagnostic implications (Haier et al., 2023; Hunt, 2011). Second, the field of intelligence has played a central role in the development of modern psychometrics. This makes it especially relevant to examine whether current tests maintain high standards of documentation, validity, reliability, and norms (Abad, 2024; Haier et al., 2023). Third, comparison with tests of other constructs makes it possible to determine whether intelligence tests show a differential quality profile or share strengths and weaknesses with the broader set of instruments reviewed by the CNT (Guilera & Barrios, 2025). This comparison is especially informative because intelligence tests tend to rest on more consolidated theoretical frameworks than other psychological constructs, which makes it possible to anticipate whether this theoretical consolidation also translates into better technical documentation.

This domain comparison, however, requires accounting for the evaluation cohort. Prior CNT-based research has shown that more recently evaluated tests tend to report more information—for example, on differential item functioning or temporal stability—even when this does not translate into significantly higher quality scores (Guilera & Barrios, 2025). Because psychometric reporting standards have evolved across the project’s 13 editions in this way, any apparent quality advantage of intelligence tests over tests of other constructs could, in principle, reflect a cohort artifact rather than a genuine domain effect, if intelligence tests happened to be evaluated, on average, in more recent editions. Establishing whether publisher and evaluation cohort independently account for quality differences is therefore a necessary complement to the domain comparison itself.

As noted at the outset, the present study pursues three specific objectives: (1) to analyze the psychometric and technical quality of the intelligence tests evaluated in the project across its 13 editions; (2) to compare this quality with that of instruments measuring other psychological constructs; and (3) to examine the impact of the publisher and the temporal evaluation cohort on the quality of intelligence tests (Guilera & Barrios, 2025).

The three objectives outlined above respond to a double motivation. From an applied perspective, they seek to offer practitioners a synthesized, standardized view for selecting intelligence tests according to empirical quality criteria. From a scientific perspective, they seek to draw on an accumulated corpus of independent evaluations to examine patterns of quality, temporal change, and publisher heterogeneity in one of the most relevant domains of psychological measurement (Evers et al., 2013; Recio-Saboya & Escorial, 2026).

## 2. Materials and Methods

### 2.1. Data Sources

The corpus for this study was drawn from the technical reports produced across the 13 editions of the project *Evaluation of Tests Published in Spain*. This project has been directed since 2010 by the National Test Commission (Comisión Nacional de Test [CNT]) of the Spanish General Council of Psychology (*Consejo General de la Psicología de España*). The reports systematically evaluate 115 instruments: 37 intelligence tests and 78 tests of other psychological and educational constructs. The unit of analysis is therefore the evaluation report for each instrument, not the original test manual.

Instruments were included if they met four criteria: (a) an official, publicly available CNT report; (b) assessment of cognitive abilities or other relevant psychological or educational variables; (c) evaluation using the CET or the CET-R; and (d) a global scoring structure suitable for analysis. Instruments with unpublished preliminary reports were excluded, as were instruments used only as partial examples in methodological papers without a complete report.

### 2.2. Procedure

Report preparation followed a peer-review protocol that remained stable across all 13 editions. In each edition, publishers represented on the CNT proposed the tests to be evaluated; non-commercial instruments selected directly by the Commission were occasionally added.

The CNT first appointed a coordinator for each edition, who then assigned two reviewers per test: one psychometrics expert and one expert in the instrument’s substantive content. Reviewer assignment sought geographic diversity, gender balance, and the absence of conflicts of interest. Each reviewer independently completed the corresponding CET or CET-R, generating quantitative scores and qualitative comments for each test.

The coordinator then integrated both evaluations, resolved discrepancies between reviewers, and drafted a preliminary report. Inter-rater reliability, reported across several editions of the project, has consistently been acceptable (Abad, 2024; Ponsoda & Hontangas, 2013). In the most recent edition, for example, the intraclass correlation coefficient (ICC) reached 0.84, 95% CI [0.79, 0.88] (Escorial, 2026), a value that reflects excellent agreement between independent reviewers according to Koo and Li’s (2016) criteria.

**Figure 1:**
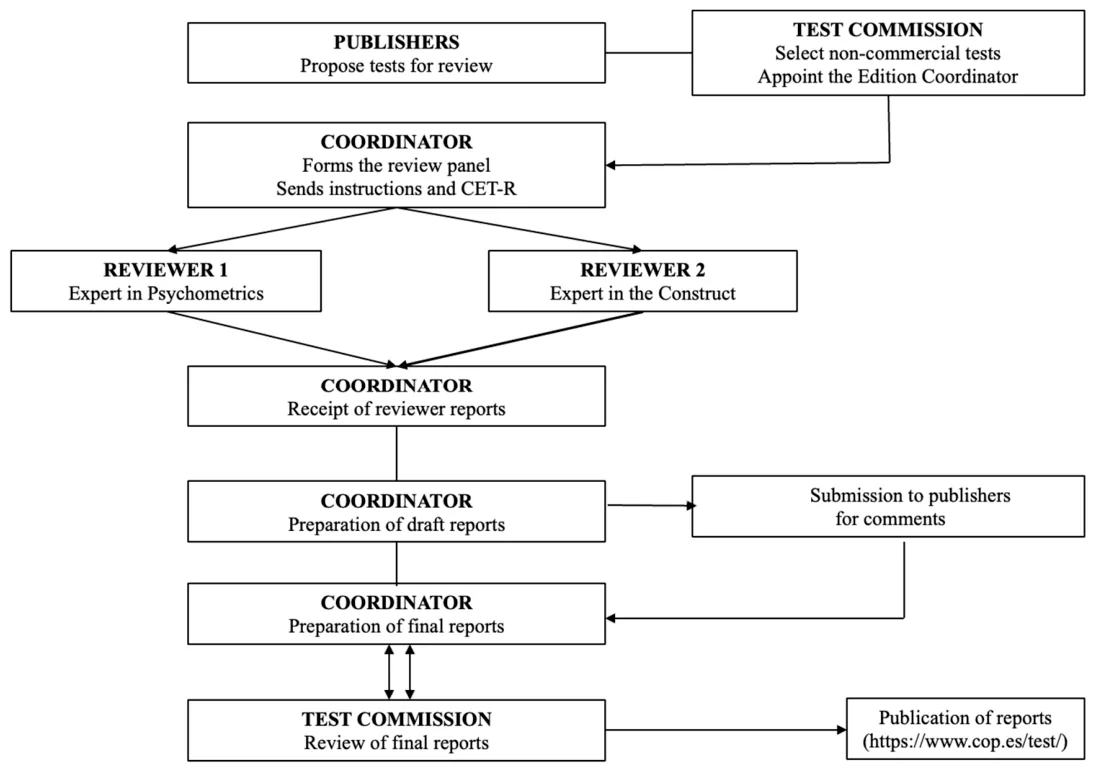
Test Evaluation Procedure Using the CET-R. ***Note.*** The diagram summarizes the phases of the annual test evaluation procedure using the CET-R: (a) test selection, (b) appointment of coordinator and reviewers, (c) independent completion of the questionnaire, (d) integration of reports, (e) publisher appeals, and (f) publication of the final report.

The preliminary report was then sent to the publishers and, where applicable, to the test authors, to gather their input. After considering the responses received, the final report was drafted. A CNT member reviewed this report prior to publication, and the final report was ultimately published on the website of the National Test Commission of the Spanish General Council of Psychology. Figure 1 summarizes these six stages: test selection, appointment of reviewers, independent completion of the CET-R, integration of reports, publisher appeals, and publication of the final report.

### 2.3. Classification of Measurement Instruments

The analyses distinguished between two groups of instruments: intelligence measures and measures of other constructs. This classification combined information from the manuals and reports with conceptual criteria established in the psychometric literature. An instrument was considered an intelligence measure when it simultaneously met the following four criteria:

1. It explicitly identified the construct assessed as intelligence or cognitive ability, consistent with its conceptualization as a general capacity to reason, solve problems, and learn from experience (Haier et al., 2023; Schneider & McGrew, 2012).
2. Its primary purpose was to assess intellectual ability—diagnosis, neuropsychological evaluation, educational guidance, or personnel selection—rather than the acquisition of curricular content or specific competencies. This criterion follows the classic distinction between aptitude tests and achievement or performance tests (AERA et al., 2014).
3. Its content focused on cognitive tasks—inductive or deductive reasoning, problem solving, working memory, visuospatial processing, or processing speed—that were, for the most part, curriculum-neutral.
4. It interpreted performance normatively, in terms of mental ability (intelligence or aptitude quotients or standard scores), rather than as academic achievement norms or psychological trait scales. This criterion follows the EFPA model and the *Standards for Educational and Psychological Testing* (AERA et al., 2014; Evers et al., 2013).

In ambiguous cases, such as certain mixed batteries, the stated objective and scoring structure of each instrument were reviewed. Only tests whose interpretive core centered on indices of intellectual ability were classified as intelligence measures. Instruments that did not meet these four criteria were grouped as measures of other constructs, a category that includes personality questionnaires, clinical scales, adaptive behavior measures, and reading and writing tests, among others. Table S1 in the Supplemental Materials provides a detailed description of the intelligence tests included in this study.

This classification, like any categorical boundary applied to a continuous and multidimensional construct, involves an element of judgment. The distinction between aptitude and achievement tests is not absolute: many aptitude batteries, including several instruments in the present sample, incorporate performance-based tasks, and the two categories are better understood as differing in emphasis and intended use rather than in kind (AERA et al., 2014). Reading and writing tests illustrate this tension directly. They were classified here as measures of other constructs because their primary purpose and normative interpretation are academic and curriculum-related rather than general cognitive ability; however, these instruments typically share substantial variance with general intelligence (*g*), consistent with the well-documented overlap between cognitive and academic skills (Haier et al., 2023). An alternative classification could have grouped instruments by their statistical relationship to g rather than by their stated purpose, but this approach was judged less suitable here, since it would conflate the empirical question this study investigates—whether documentation quality differs by construct—with the classification criterion itself. The four-criterion scheme adopted was therefore designed to prioritize a test’s declared purpose and normative framework, consistent with the EFPA model and the *Standards* (AERA et al., 2014; Evers et al., 2013), while acknowledging that borderline cases, such as reading and writing tests, could reasonably be classified otherwise under different theoretical priorities.

### 2.4. Instruments

The central instrument of this study is the Test Review Questionnaire-Revised (*Cuestionario de Evaluación de Tests Revisado*; CET-R). This questionnaire is based on the test evaluation model of the European Federation of Psychologists’ Associations (EFPA) and on international measurement standards (Evers et al., 2013; Hernández et al., 2016). The CNT has applied it systematically since the fifth edition of the project, replacing the original CET developed by Prieto and Muñiz (2000).

The CET-R is organized into three sections. The first provides a general description of the test. The second assesses the test’s psychometric characteristics. The third provides an overall evaluation in the form of a conclusion. Quantitative responses use a 1-to-5 scale with the following ratings: 1 (*Inadequate*), 2 (*Adequate but with shortcomings*), 3 (*Adequate*), 4 (*Good*), and 5 (*Excellent*). Some individual items also include an additional not-applicable (N/A) category, which covers both information that was not reported in the manual and information that does not apply given the characteristics and purpose of the specific test being evaluated.

The general test description comprises 28 items: authorship, publication dates, target population, format, administration conditions, required examiner qualifications, and price, among others. This section does not yield scores.

The assessment of psychometric characteristics constitutes the core section of the CET-R and is organized into four grouped dimensions. The General dimension (10 items) gathers evidence on the quality of materials and documentation, theoretical foundation, adaptation, and item analysis and test development. The Validity dimension (19 items) covers content validity, relations with other variables, internal structure, and differential item functioning. The Reliability dimension (14 items) addresses parallel-forms equivalence, internal consistency, temporal stability, item response theory (IRT) based reliability, and inter-rater reliability. The Norms dimension (9 items) gathers information on the representativeness and currency of the norms, continuous norming, and cutoff scores. Each grouped dimension also includes an open-ended item in which reviewers justify their ratings.

The overall evaluation integrates a narrative report describing the test’s strengths and weaknesses, along with recommendations for use and suggested improvements. It also includes global scores for the CET-R’s 14 main individual characteristics.

### 2.5. Coding Procedure

This study extracted the aggregate scores reported for the 14 individual characteristics in the CET-R’s Overall Evaluation section. Reports from the earliest editions were based on the original CET rather than the CET-R; their scores were recoded into the CET-R’s 14 characteristic framework following the guidelines established in previous studies (Guilera & Barrios, 2025).

The 14 individual characteristics were organized into four grouped dimensions: General (materials, theoretical foundation, adaptation, and item analysis), Validity (content, relations with other variables, internal structure, and differential item functioning), Reliability (equivalence, internal consistency, stability, IRT, and inter-rater reliability), and Norms. A Global Score was also computed from the 14 individual characteristics. When an individual characteristic was not applicable, it was excluded from the analyses of its corresponding grouped dimension and from the Global Score.

### 2.6. Statistical Analysis

All analyses were conducted in RStudio (Version 2026.07.1+147; Posit Team, 2026). Data manipulation and cleaning used *tidyverse*. Base R statistical functions were supplemented with the *car*, *psych*, and *effectsize* packages. Visualization was performed with *ggplot2* and *corrplot*. The significance level was set at α = 0.05 for all analyses.

The first analytic phase focused on the missing-data rate at the level of the individual characteristic. Variables were treated dichotomously: a score available on the 1-to-5 scale versus an N/A code (information not reported in the manual, or not applicable given the test’s characteristics and purpose). This analysis considered 12 of the 14 individual characteristics in the Overall Evaluation section. Two characteristics were excluded: Equivalence between parallel forms, owing to its limited applicability, and Adaptation, because an N/A code for this characteristic indicates that the test was originally developed in Spain rather than reflecting an actual omission of information. Frequencies and omission percentages were calculated for each individual characteristic.

Differences in the rate of information inclusion between intelligence tests and tests of other constructs were tested using Fisher’s exact test, with the magnitude of the association estimated via Cramér’s *V*. In addition, the nonparametric Mann–Whitney *U* test was used to compare overall percentages of missing data grouped by dimension; this analysis treated each test’s report as the unit of analysis (*N* = 37 vs. 78). Effect size was quantified using the *r* statistic. Given the pronounced skew in omission percentages, the median and interquartile range (IQR) of each grouped dimension were also reported.

The second analytic phase focused on the technical quality of the reported information, both at the level of the individual characteristic and of the grouped dimension (General, Validity, Reliability, Norms, and Global Score). This phase was restricted to individual characteristics with available information—that is, cases not included in the missing-data analysis of the first phase. Scores analyzed in this phase therefore fall within the CET-R’s full 1-to-5 scale. Welch’s *t*-tests for independent samples were applied; these tests do not assume homogeneity of variance across groups and adjust degrees of freedom using Satterthwaite’s approximation. Intelligence measures were compared against measures of other constructs, and the magnitude of the differences was estimated using Hedges’ *g*, calculated from each group’s unpooled standard deviations.

Given the modest sample size of the intelligence-test group (*N* = 37), a post hoc sensitivity (power) analysis was additionally conducted for all 17 comparisons reported in Table S2. For each comparison, we computed (a) the observed statistical power to detect the effect size (Hedges’ *g*) actually obtained, and (b) the minimum detectable effect size (MDE, expressed as Hedges’ *g*) that the available sample sizes could identify with 80% power at α = 0.05 (two-tailed), using the *pwr* package (Champely, 2020) in R. This analysis makes it possible to interpret non-significant comparisons in light of the magnitude of effect the design was actually capable of detecting, rather than treating non-significance as evidence of an absent difference. Observed Power and MDE values for each comparison are reported alongside the corresponding Welch’s *t*-test results in Table S2.

To examine the effect of commercial publisher and edition cohort on the quality of intelligence measures, the nonparametric Kruskal–Wallis *H* test was used. This test was appropriate given violations of normality and homoscedasticity, as well as the small sample sizes of some subgroups. The proportion of variance explained was quantified using two statistics: rank-based eta-squared (η^2^_H_) and epsilon-squared (ε^2^).

Given the large number of comparisons performed, false discovery rate (FDR) control was applied (Benjamini & Hochberg, 1995), separately within each family of tests: missing data, quality comparisons, publisher effects, and cohort effects. This procedure was preferred over the Bonferroni correction because of its lower risk of Type II error with small sample sizes. Both uncorrected and FDR-adjusted *p*-values are reported.

Finally, the performance of each instrument was represented using heatmaps, both at the level of the individual characteristic and the grouped dimension, including the Global Score. This visual analysis made it possible to identify profiles of excellence and areas of vulnerability among the tests evaluated.

To statistically substantiate the visual impression of within-test consistency conveyed by these heatmaps, the internal consistency of each test’s four-dimension quality profile (General, Validity, Reliability, Norms) was additionally quantified using Cronbach’s alpha and the consistency form of the intraclass correlation coefficient, ICC(C,k) (two-way mixed-effects model, dimensions as fixed raters), computed among the 37 intelligence tests with complete data across all four dimensions. As a complementary exploratory approach, the correlational structure underlying this profile was also examined through a single-factor factor analysis (principal axis factoring), following Kaiser-Meyer-Olkin and Bartlett’s sphericity checks of factorability. Because *N* = 37 falls below sample sizes typically recommended for factor analysis, this result is interpreted tentatively, as a descriptive complement to alpha and ICC rather than as confirmatory evidence of the factor structure.

## 3. Results

The analysis was organized into two phases. The first assessed the amount of information reported in the manuals, comparing missing-data rates between intelligence tests and tests of other constructs. The second assessed the quality of that information, only when it was available, and examined the effect of publisher and evaluation cohort on the quality of intelligence tests. A final visual analysis, using heatmaps, summarized each instrument’s individual psychometric profile, both at the level of the 14 individual characteristics and the 4 grouped dimensions (General, Validity, Reliability, and Norms).

### 3.1. Patterns of Missing Data

The analysis excluded the characteristic of equivalence of parallel forms. This characteristic applied to very few instruments: only 5.41% of intelligence tests (2 of 37) and 3.85% of tests of other constructs (3 of 78) reported it. Its exclusion therefore reflected its limited applicability, not a documentation problem.

**Figure 2:**
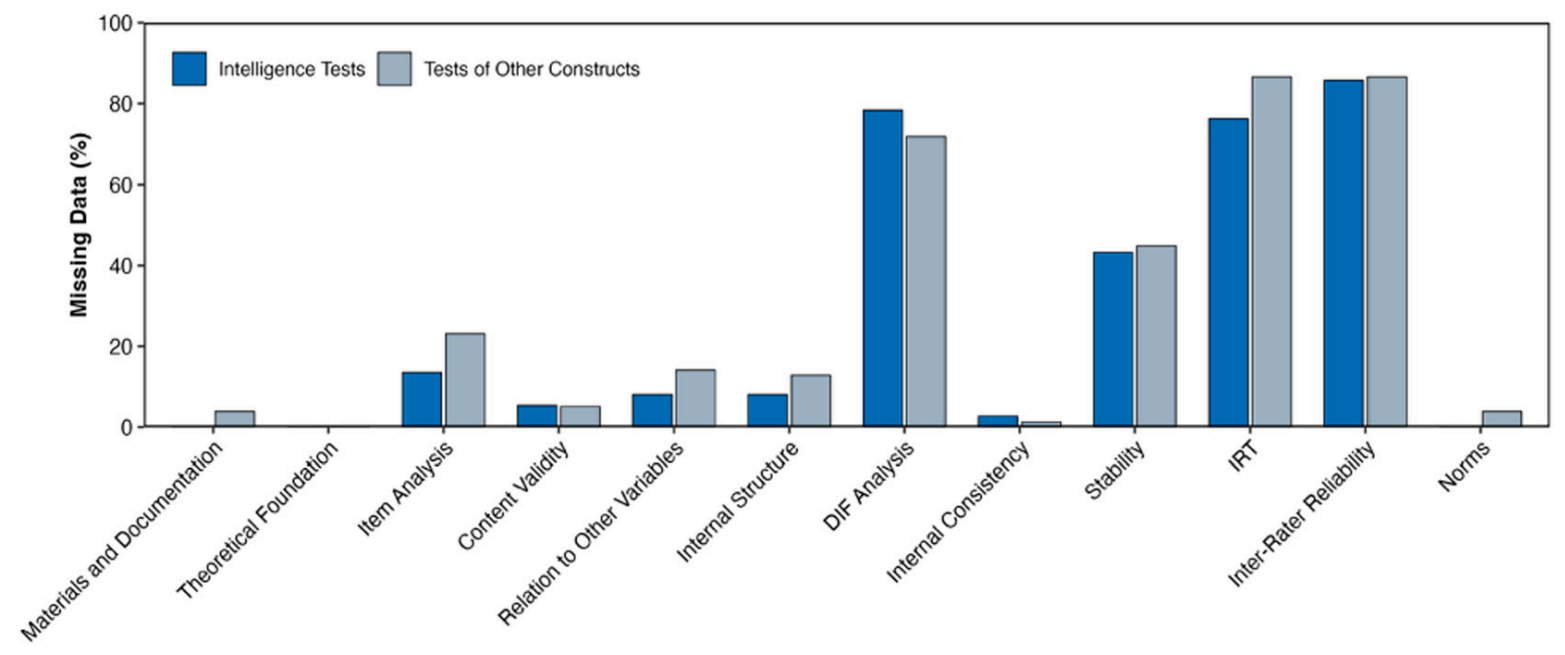
Percentage of Missing Data Across Individual Psychometric Characteristics in Intelligence Tests and Tests of Other Constructs. ***Note.*** Percentage of missing data (unreported or non-evaluable information in test manuals) for each of the 12 individual characteristics evaluated with the CET-R. Dark blue bars represent intelligence tests; light grey bars represent tests of other psychological constructs. The Equivalence characteristic was excluded due to widespread data unavailability. Analyses for IRT (Item Response Theory) and Inter-Rater Reliability were restricted to test editions published in 2016 or later.

The remainder of the analysis temporarily adjusted for the evaluation cohort. This adjustment ensured comparability for CET-R characteristics of recent introduction, such as reliability estimated through Item Response Theory (IRT) and inter-rater reliability, systematically evaluated only since 2016. With this adjustment, results showed no significant differences in the amount of omitted information between the two groups of tests (*p* ≥ .311, *p*_FDR_ ≥ 0.808; Cramer’s *V* ≤ 0.13). Figure 2 shows the omission percentage for each individual characteristic, comparing the group of intelligence measures with the group of measures of other constructs.

The analysis by individual characteristic (Figure 2) revealed a similar reporting pattern in both types of tests. Theoretical foundation, materials and documentation, norms, and internal consistency were documented almost universally, with omission rates below 4% in both groups. By contrast, differential item functioning (DIF) analysis, temporal stability, IRT-based reliability, and inter-rater reliability showed substantial omissions in both groups, the latter two—required only since 2016—reaching omission rates above 76% and 85%, respectively, with no differences between test types.

When grouped by dimension (Table 1), none of the four grouped dimensions or the Global Score showed a statistically significant difference in omission rates between groups (all *p* ≥ .111, *p_FDR_* ≥ 0.288). Reliability accounted for the largest share of missing information in both groups, with a median omission rate around 50%, while Validity showed an identical median omission rate in both groups (25.0%).

### 3.2. Psychometric Quality of Reported Information

**Figure 3:**
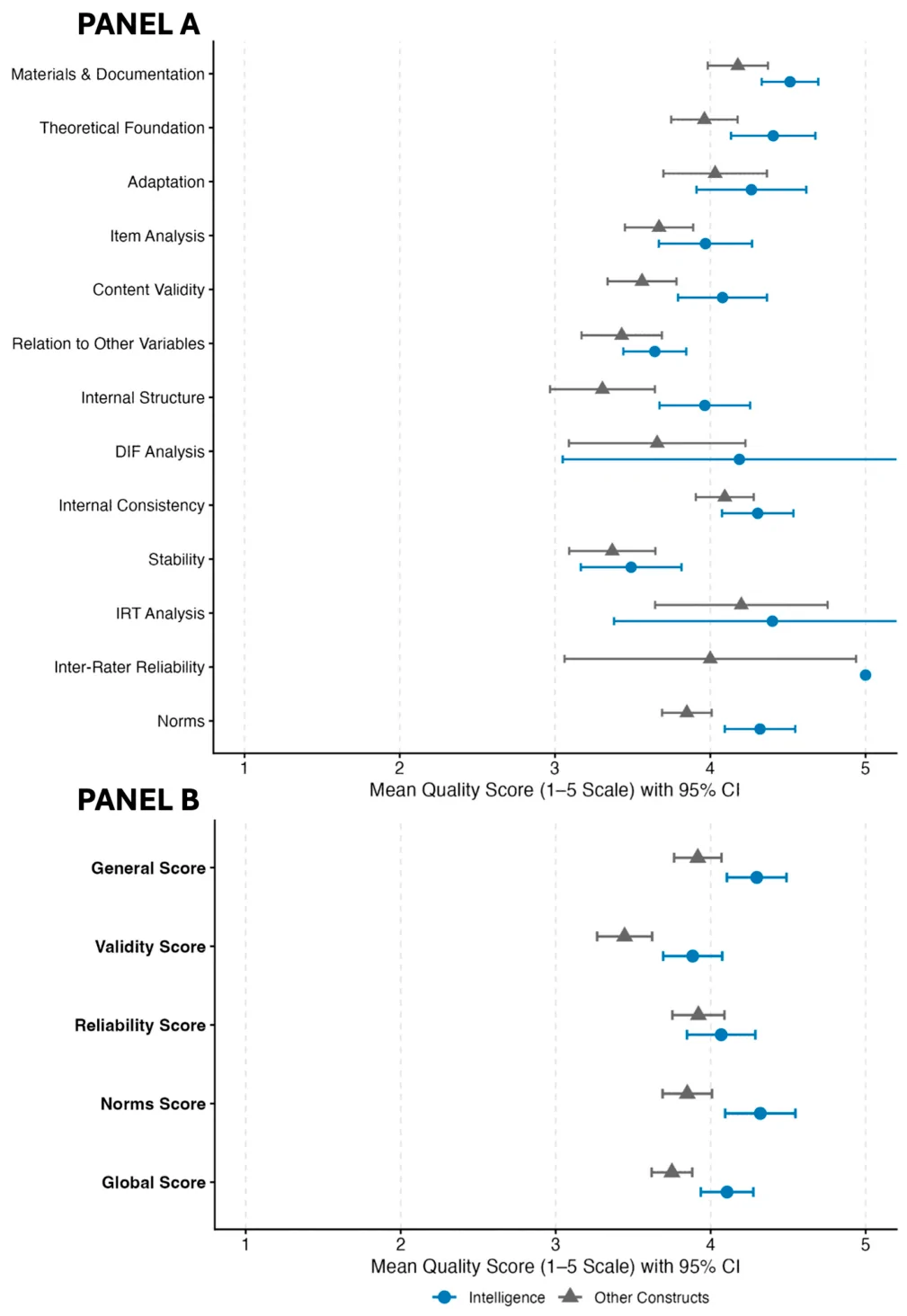
Comparison of Psychometric Quality Scores Between Intelligence Tests and Tests of Other Constructs Across Individual Characteristics and CET-R Dimensions. ***Note.*** (**Panel A**) shows mean quality scores and 95% confidence intervals (CIs) for the evaluated individual psychometric characteristics. (**Panel B**) presents aggregated mean scores for the General, Validity, Reliability, and Norms dimensions, as well as the Global Score. All characteristics and dimensions are evaluated on a 1–5 scale, where higher scores reflect higher psychometric quality. Error bars represent 95% CIs. Sample sizes (*n*) vary across characteristics due to available-case analysis.

The second phase analyzed the quality of the available information, on the CET-R’s 1-to-5 scale. This analysis compared scores for intelligence tests (N = 37) with scores for tests of other constructs (N = 78). Figure 3 details this comparison.

Intelligence tests obtained significantly higher quality on the Global Score (*M* = 4.11, *SD* = 0.51) than tests of other constructs (*M* = 3.75, *SD* = 0.58), Welch’s *t*(80.23) = 3.35, *p* = .001, *p_FDR_* = 0.006, Hedges’ *g* = 0.65. As Panel B of Figure 3 shows, this advantage was also present in three of the four dimensions evaluated.

The Validity dimension also showed a significant difference favoring intelligence measures (*M* = 3.88, *SD* = 0.57) over measures of other constructs (*M* = 3.44, *SD* = 0.76), Welch’s *t*(92.35) = 3.40, *p* = .001, *p_FDR_* = 0.006, Hedges’ *g* = 0.65. At the individual-characteristic level (Figure 3, Panel A), content validity (*M* = 4.08, *SD* = 0.83, vs. *M* = 3.56, *SD* = 0.84; Welch’s *t*(72.51) = 2.89, *p* = .005, *p_FDR_* = 0.014, Hedges’ *g* = 0.61) and internal structure (*M* = 3.96, *SD* = 0.84, vs. *M* = 3.31, *SD* = 1.24; Welch’s *t*(85.51) = 2.98, *p* = .004, *p_FDR_* = 0.014, Hedges’ *g* = 0.62) showed significant advantages. Relation to other variables did not reach statistical significance (*M* = 3.64, *SD* = 0.58, vs. *M* = 3.43, *SD* = 0.85; Welch’s *t*(74.97) = 1.31, *p* = .194, *p_FDR_* = 0.275, Hedges’ *g* = 0.29). Nor did DIF analysis show significant differences (*M* = 4.19, *SD* = 1.36, vs. *M* = 3.66, *SD* = 1.18; Welch’s *t*(11.67) = 0.96, *p* = .357, *p_FDR_* = 0.405, Hedges’ *g* = 0.39); this last result requires caution, given the small number of manuals reporting this analysis in both groups.

On the Norms dimension, intelligence measures also obtained significantly higher quality (*M* = 4.32, *SD* = 0.68, vs. *M* = 3.85, *SD* = 0.63), Welch’s *t*(71.27) = 3.43, *p* = .001, *p_FDR_* = 0.006, Hedges’ *g* = 0.71.

The General dimension likewise showed significant differences (*M* = 4.30, *SD* = 0.58, vs. *M* = 3.92, *SD* = 0.66), Welch’s *t*(81.46) = 3.12, *p* = .003, *p_FDR_* = 0.013, Hedges’ *g* = 0.61). This advantage was statistically significant for two of its four constituent characteristics, materials and documentation (*M* = 4.51, *SD* = 0.55, vs. *M* = 4.18, *SD* = 0.74; Welch’s *t*(90.81) = 2.54, *p* = .013, *p_FDR_* = 0.028, Hedges’ *g* = 0.51) and theoretical foundation (*M* = 4.41, *SD* = 0.82, vs. *M* = 3.96, *SD* = 0.87; Welch’s *t*(78.82) = 2.58, *p* = .012, *p_FDR_* = 0.028, Hedges’ *g* = 0.52). Adaptation (*M* = 4.26, *SD* = 0.69, vs. *M* = 4.03, *SD* = 0.92; Welch’s *t*(41.65) = 1.00, *p* = .323, *p_FDR_* = 0.392, Hedges’ *g* = 0.28) and item analysis (*M* = 3.97, *SD* = 0.83, vs. *M* = 3.67, *SD* = 0.77; Welch’s *t*(62.63) = 1.63, *p* = .108, *p_FDR_* = 0.184, Hedges’ *g* = 0.37) did not reach statistical significance individually, although both likewise showed higher mean scores for intelligence tests (Figure 3, Panel A) and therefore also contributed quantitatively to the aggregate dimension score.

The Reliability dimension, by contrast, showed no differences between the two groups of instruments (*M* = 4.07, *SD* = 0.66, vs. *M* = 3.92, *SD* = 0.69), Welch’s *t*(77.18) = 1.07, *p* = .288, *p_FDR_* = 0.377, Hedges’ *g* = 0.21. None of its individual components reached statistical significance: neither internal consistency (*M* = 4.31, *SD* = 0.68, vs. *M* = 4.09, *SD* = 0.72; Welch’s *t*(77.45) = 1.45, *p* = .152, *p_FDR_* = 0.235, Hedges’ *g* = 0.30), nor stability (*M* = 3.49, *SD* = 0.71, vs. *M* = 3.37, *SD* = 0.77; Welch’s *t*(45.28) = 0.59, *p* = .559, *p_FDR_* = 0.594, Hedges’ *g* = 0.16), nor IRT (*M* = 4.40, *SD* = 0.82, vs. *M* = 4.20, *SD* = 0.45; Welch’s *t*(6.18) = 0.48, *p* = .649, *p_FDR_* = 0.649, Hedges’ *g* = 0.26). The sample size for this last characteristic was extremely small (*n* = 5 in each group), which limits any conclusion. Inter-rater reliability did show a significant difference favoring intelligence measures (*M* = 5.00, *SD* = 0.00, *n* = 3, vs. *M* = 4.00, *SD* = 0.89, *n* = 6; Welch’s *t*(5.00) = 2.74, *p* = .041, *p_FDR_* = 0.077, Hedges’ *g* = 1.33), but this difference disappears after FDR correction. Its minimal sample size and the total absence of variability in the intelligence group prevent it from being considered a stable estimate.

A sensitivity analysis further clarifies the pattern of (non-)significant findings reported above (Table S2). Across the eight comparisons that reached statistical significance, observed effect sizes were consistently large enough to exceed the sample-specific detection threshold (Hedges’ *g* ≥ 0.51, against MDEs of approximately 0.56–0.62 at 80% power). By contrast, the nine comparisons that did not reach significance—Adaptation, Item Analysis, Relation to Other Variables, DIF Analysis, Internal Consistency, Stability, IRT Analysis, the Reliability Score, and Inter-Rater Reliability—showed observed effect sizes below their corresponding MDE (Hedges’ *g* = 0.16–0.39, versus MDEs of 0.58–2.31; the single exception, Inter-Rater Reliability, showed a large observed effect, *g* = 1.33, but with an MDE of 2.31 owing to its extremely small sample). This indicates that, with the exception of Stability (*g* = 0.16, close to a null effect), the present design lacked adequate power to rule out small-to-moderate differences in these characteristics, and the absence of statistical significance should not be interpreted as evidence that intelligence tests and tests of other constructs are equivalent on these characteristics.

In sum, intelligence tests stand out in validity, norms, materials, and theoretical foundation. On reliability, they maintain a level similar to tests of other constructs. Table S2 in the Supplementary Materials provides full detail on these comparisons.

### 3.3. Publisher and Evaluation Cohort Effects

CET-R reports also record the publisher and the year in which the CNT evaluated each instrument. This made it possible to examine whether these two factors account for part of the quality observed within the group of intelligence measures. Given the ordinal nature of the scores, their skewed distribution, and the small size of some subsamples, the non-parametric Kruskal–Wallis test was used. Effect size was estimated using two statistics: rank-based eta-squared (η^2^H) and epsilon-squared (ε^2^). All analyses excluded the equivalence characteristic, due to its previously noted lack of data, and inter-rater reliability, which could not be evaluated inferentially due to insufficient sample size.

**Figure 4:**
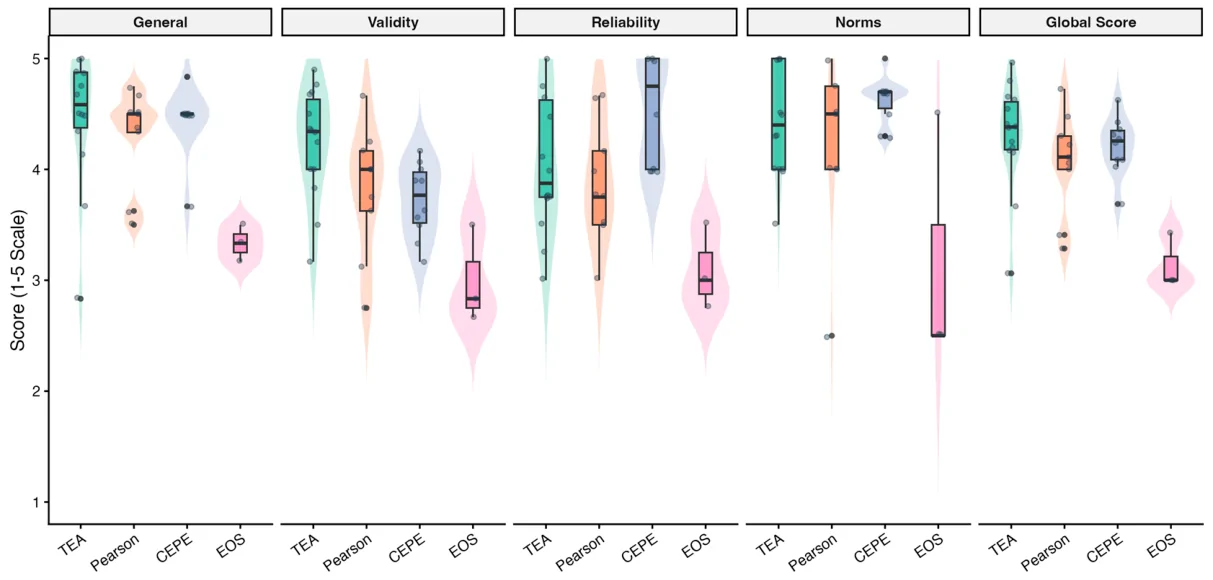
Distribution of Psychometric Quality Scores by Publisher and Evaluated Dimension. ***Note.*** Color indicates publisher and is consistent across the five panels: TEA-Hogrefe is shown in green, Pearson in orange, CEPE in blue, and EOS in pink; publisher identity is also labeled on the x-axis of each panel. Hybrid violin plots and boxplots with overlaid individual data points illustrate the distribution of quality evaluation scores (1–5 scale) across the four main CET-R dimensions and the Global Score, by publisher. Within the boxplots, the horizontal line represents the median, the box bounds denote the interquartile range (IQR; 25th to 75th percentiles), and the whiskers extend up to 1.5 × IQR. The violin shapes reflect the kernel density estimation of the score distribution.

The analysis of publisher effect excluded the group’s only non-commercial test (N = 36). The distribution of tests by publisher was uneven: TEA-Hogrefe (*n* = 14), Pearson (*n* = 9), CEPE (*n* = 10), and EOS (*n* = 3). Publisher accounted for a substantial share of the variance in the Global Score, *H*(3) = 9.48, *p* = .024, *p_FDR_* = 0.038, η^2^H = 0.20, ε^2^ = 0.27. This pattern was repeated for Validity, *H*(3) = 12.44, *p* = .006, *p_FDR_* = 0.019, η^2^H = 0.30, ε^2^ = 0.36, for Reliability, *H*(3) = 12.41, *p* = .006, *p_FDR_* = 0.019, η^2^H = 0.29, ε^2^ = 0.35, and for the General dimension, *H*(3) = 9.22, *p* = .026, *p_FDR_* = 0.038, η^2^H = 0.19, ε^2^ = 0.26. Norms, by contrast, showed no differences between publishers, *H*(3) = 4.97, *p* = .174, *p_FDR_* = 0.197, η^2^H = 0.06, ε^2^ = 0.14. Figure 4 illustrates this comparison.

The analysis by individual characteristic confirmed substantial inequalities between publishers. Within the General dimension, differences stood out for materials and documentation, *H*(3) = 15.97, *p* = .001, *p_FDR_* = 0.016, η^2^H = 0.41, ε^2^ = 0.46, item analysis, *H*(3) = 10.16, *p* = .017, *p_FDR_* = 0.037, η^2^H = 0.27, ε^2^ = 0.34, and theoretical foundation, *H*(3) = 9.99, *p* = .019, *p_FDR_* = 0.037, η^2^H = 0.22, ε^2^ = 0.29. On Validity, differences were especially marked for relation to other variables, *H*(3) = 14.08, *p* = .003, *p_FDR_* = 0.016, η^2^H = 0.38, ε^2^ = 0.44, internal structure, *H*(3) = 13.90, *p* = .003, *p_FDR_* = 0.016, η^2^H = 0.36, ε^2^ = 0.42, content validity, *H*(3) = 10.96, *p* = .012, *p_FDR_* = 0.032, η^2^H = 0.26, ε^2^ = 0.32, and DIF analysis, *H*(1) = 5.33, *p* = .021, *p_FDR_* = 0.037, η^2^H = 0.72, ε^2^ = 0.76. On Reliability, only internal consistency showed a publisher effect, though it disappears after FDR correction, *H*(3) = 8.04, *p* = .045, *p_FDR_* = 0.060, η^2^H = 0.16, ε^2^ = 0.24. Table S3 in the Supplementary Materials provides full detail on these comparisons.

The effect of evaluation cohort was examined across 11 different years (N = 37). No dimension showed a significant trend: neither General, Validity, Reliability, Norms, nor the Global Score (*p* ≥ .071, *p_FDR_* ≥ 0.208). When each characteristic was analyzed separately, only theoretical foundation, *H*(10) = 22.16, *p* = .014, *p_FDR_* = 0.208, η^2^H = 0.47, ε^2^ = 0.62, and internal structure, *H*(10) = 18.90, *p* = .042, *p_FDR_* = 0.208, η^2^H = 0.39, ε^2^ = 0.57, showed a cohort effect in their uncorrected *p* value; both effects disappear after FDR correction. The remaining characteristics showed no significant temporal variation. DIF analysis could not be evaluated by cohort: only 8 manuals (21.6% of the sample) reported it, spread across seven different years, so the minimum sample size per group was not met. This characteristic is therefore described qualitatively, as a practice that remains uncommon in the evaluation of intelligence tests. Table S4 in the Supplementary Materials provides full detail on these comparisons.

### 3.4. Test-Level Psychometric Profiles

**Figure 5:**
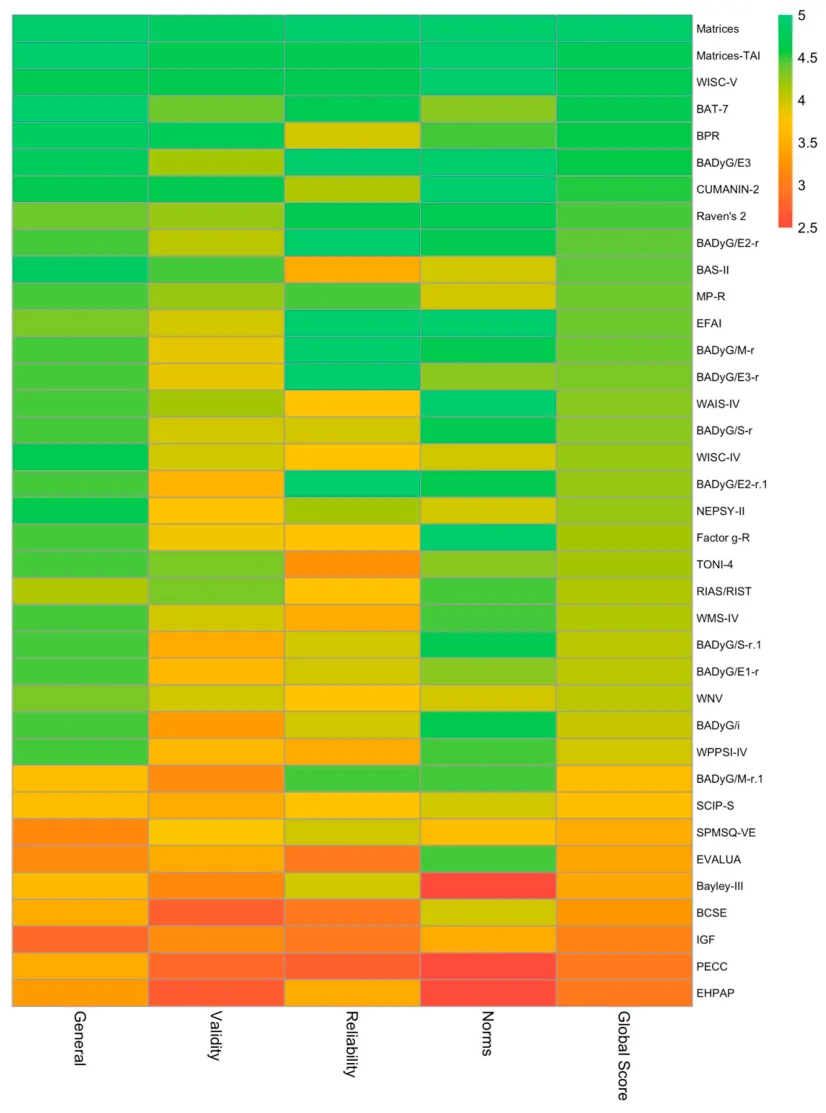
Heatmap of Dimension Scores and Global Score Across Evaluated Tests. ***Note.*** Visual heatmap representation of mean psychometric quality scores (1–5 scale) across the four main CET-R dimensions (General, Validity, Reliability, and Norms) and the Global Score for each evaluated test. Color gradients range from green tones (maximum quality, 5.0) to red tones (low quality, 2.5). Tests are arranged hierarchically based on their overall performance.

Finally, quality scores were represented using heatmaps, to jointly examine each instrument’s psychometric profile. Unlike the CNT’s annual reports, which present the results of a single evaluation cohort at a time, this analysis aggregates data across all 13 editions (2010–2026) into a single comparative visualization, ranking tests by overall performance regardless of the year in which they were evaluated. Figure 5 shows this analysis at the grouped-dimension level; Figure S1 in the Supplementary Materials shows it at the individual-characteristic level.

At the dimension level (Figure 5), psychometric quality tends to be consistent within each instrument. Tests with strong overall performance show high scores on General, Validity, Reliability, and Norms characteristics simultaneously. Tests with lower overall quality, by contrast, show weaknesses across several dimensions at once. Instruments such as Matrices, Matrices-TAI, WISC-V, BAT-7, and CUMANIN-2 maintain excellent scores (≥4 points) across nearly all dimensions. Tests such as PECC, EHPAP, IGF, and Bayley-III, in contrast, show areas for improvement across several dimensions (≤3 points).

This visual impression of consistency was corroborated statistically. Cronbach’s alpha across the four dimension scores was α = 0.84, 95% CI [0.74, 0.91]—mathematically equivalent to the average-measures intraclass correlation coefficient, ICC(C,4) = 0.84, 95% CI [0.73, 0.91]—indicating that a test’s standing on one dimension is a good predictor of its standing on the others. Pairwise correlations among dimensions ranged from moderate to strong (*r* = 0.45–0.75), with Reliability showing the weakest, though still moderate, associations with the remaining three dimensions (*r* = 0.45–0.51).

As an exploratory approach, a single-factor factor analysis (KMO = 0.78; Bartlett’s χ^2^(6) = 60.95, *p* < .001) was consistent with a common quality factor underlying the four dimensions, accounting for 59–69% of their shared variance (loadings: General = 0.86, Validity = 0.83, Norms = 0.76, Reliability = 0.59; Reliability communality = 0.35). This result should be interpreted with caution: with only 37 cases and four variables, the RMSEA confidence interval was wide (90% CI [0, 0.29]), and the likelihood-ratio test of whether one factor is sufficient, although non-significant (χ^2^(2) = 1.33, *p* = 0.51), has limited power at this sample size to rule out alternative structures. This factor analysis is therefore presented as a descriptive complement to alpha and ICC rather than as confirmatory evidence of the factor structure, and its replication in larger samples remains a useful direction for future research. Taken together, both analyses—the more robust reliability indices and the exploratory factor analysis—converge on the same conclusion: psychometric quality is not distributed independently across dimensions within a given intelligence test, while also showing that Reliability retains a somewhat more distinct profile, consistent with its comparatively idiosyncratic behavior already reported in Section 3.2.

The analysis at the individual-characteristic level (Figure S1 in Supplemental Material) provides further detail on these differences. Materials and documentation and theoretical foundation receive high scores across most tests. DIF analysis, IRT-based reliability, and relation to other variables, by contrast, show the greatest vulnerabilities: many empty cells or low scores, especially among tests of medium or low overall quality.

## 4. Discussion

These results make it possible to assess the metric quality of the intelligence tests evaluated by the National Test Commission (Comisión Nacional de Test [CNT]). Intelligence tests do not provide more psychometric information than tests of other constructs; both groups omit a similar percentage of CET-R indicators. When information is reported, intelligence tests reach higher quality on the General, Validity, and Norms dimensions. Publisher accounts for much of this heterogeneity. Evaluation cohort, by contrast, does not.

Beyond their applied value for test selection, these findings offer three contributions of scientific interest to psychology. First, they provide an empirical test—rather than a purely theoretical argument—of the hypothesis that a construct’s theoretical consolidation translates into better technical documentation. Second, they demonstrate that systematically coded institutional test-review reports constitute a viable, underused data source for the empirical study of psychometric practice, a methodological approach that could be extended to other national review systems built on the EFPA model. Third, the profile-consistency analysis presented in Section 3.4 offers new evidence that the four CET-R dimensions are not empirically independent but instead largely reflect a single general documentation-quality factor, from which Reliability partially diverges—a finding with implications for how test-quality frameworks such as the CET-R and EFPA model are conceptualized and scored.

### 4.1. Amount of Information Versus Quality of Information

The similarity in omission rates between intelligence tests and tests of other constructs suggests that the lack of evidence is not a domain-specific problem. It is, rather, a cross-cutting limitation (Muñiz et al., 2011). This equivalence was especially marked for Validity, where both groups showed an identical median omission rate (25.0%). Information gaps, therefore, do not distinguish intelligence tests from other constructs. Moreover, the Reliability dimension accounted for the largest share of missing data in both groups, at around 50%.

This pattern matches recurring findings from previous editions of the project. Temporal stability, IRT-based reliability, inter-rater reliability, and differential item functioning (DIF) analysis have repeatedly been identified as the most neglected aspects of technical manuals (Escorial, 2026; Fonseca-Pedrero & Muñiz, 2017; Guilera & Barrios, 2025). DIF, for example, was missing in 78.38% of intelligence tests and 71.79% of the remaining tests. This figure has barely improved since the project’s earliest editions, when almost no test included this analysis (Gómez-Sánchez, 2019; Muñiz et al., 2011). A plausible explanation is economic: generating invariance evidence or calibrating an item bank through IRT requires large samples and costly longitudinal or cross-cultural studies, which are not always cost-effective for mid-sized publishers (Elosua & Geisinger, 2016; Hidalgo & Hernández, 2019).

When information is available, its quality clearly differs between groups. Intelligence tests obtained a significantly higher Global Score. This advantage on Validity, Norms, and the General dimension is consistent with the psychometric tradition of intelligence. The intelligence domain has a consolidated theoretical framework, the CHC model, widely accepted by authors and publishers (Caemmerer et al., 2020; Haier et al., 2023; Schneider & McGrew, 2012). This framework offers clear guidance for documenting theoretical foundation and designing construct-validity studies. Tests of other constructs, by contrast, often face a more fragmented theoretical landscape, with less consensus on the construct’s own definition and on which validity evidence should take priority (AERA et al., 2014; Evers et al., 2013). The advantage on norms is likewise consistent with the long normative tradition of intellectual assessment, which has adopted continuous norming techniques more quickly than other areas of psychological assessment (Abad, 2024).

The absence of differences on reliability indicates that internal consistency has become a common minimum standard for any publishable test. This convergence makes sense: classic reliability indices have been part of any psychometrician’s training for decades, regardless of the construct assessed. Reliability estimated through item response theory, by contrast, is a recent requirement that authors and publishers have not yet fully adopted (Abad, 2024; Guilera & Barrios, 2025; Recio-Saboya, 2026).

This CHC-related pattern can be illustrated with two instruments identified in Section 3.4 at opposite ends of overall performance. WISC-V, among the top-performing tests, is explicitly structured around five CHC broad abilities: Verbal Comprehension (Gc), Visual Spatial (Gv), Fluid Reasoning (Gf), Working Memory (Gwm), and Processing Speed (Gs) (Wechsler, 2014). Bayley-III, among the lowest-scoring instruments, organizes its content around developmental domains—cognitive, language, and motor functioning—rather than around a hierarchical model of cognitive abilities such as CHC (Bayley, 2006). This two-instrument contrast cannot establish a general trend across the full sample, but it is consistent with the argument developed above.

### 4.2. Publisher Effect and Absence of Cohort Effect

The significant effect of publisher on quality, with large effect sizes for Validity, Reliability, and the General dimension, suggests that each publisher’s internal policies shape an instrument’s final quality more than the passage of time does. The practical reading is direct. Some publishers consistently maintain higher standards in their technical manuals. Others show persistent shortcomings, regardless of the year in which the CNT evaluated the test. In fact, no psychometric characteristic showed a significant cohort effect once the significance level was corrected for multiple comparisons.

This finding partly contrasts with that of Guilera and Barrios (2025). These authors found that manuals from more recent cohorts qualitatively contained more information, although they likewise found no significant improvement in quantitative quality indicators across editions. Our results agree with this second conclusion: quality does not improve significantly as editions progress. We cannot confirm the first, because in our data the amount of reported information also does not vary across cohorts. This discrepancy may stem from a methodological difference: Guilera and Barrios (2025) analyzed the CNT’s entire set of evaluated tests, whereas the present study focuses only on intelligence, a domain where psychometric tradition was already strong before the evaluation project began. Either way, both results point in the same direction: the passage of time alone does not improve test quality. Improvement depends on publishers’ active decisions, possibly spurred by international concern for the quality of assessment instruments (Hernández et al., 2022).

### 4.3. Persistent Vulnerabilities

Beyond the differences between groups, the profiles obtained by intelligence tests on the CET-R dimensions (Figure 5) and individual characteristics (Figure S1) reveal vulnerabilities shared across nearly the entire sector. Differential item functioning and IRT-based reliability show the highest omission rates, well above the remaining indicators. This gap is relevant because this evidence is what guarantees measurement fairness across demographic groups and the precision of scores at the characteristic level (AERA et al., 2014). This same limitation has repeatedly been noted in previous editions of the CNT project (Fonseca-Pedrero & Muñiz, 2017; Hernández et al., 2022), confirming that it is a structural challenge for the sector, not a specific weakness of intelligence tests.

### 4.4. International Generalizability of the CET-R Model

Although this study is grounded in a specifically Spanish evaluation system, the underlying question—whether intelligence tests are documented to a standard that supports responsible use—is not a national issue. Two considerations help situate these findings within the broader international landscape of test review.

First, the CET-R is not an isolated instrument. It descends directly from the EFPA Review Model (Evers et al., 2013), which was itself built by integrating three national systems that had developed independently: the British Psychological Society’s review criteria, the Dutch COTAN rating system, and the original Spanish CET on which the CET-R is based (Evers et al., 2013). A comparative analysis of national and international review systems—covering the US Buros Center for Testing, the German Test Review System, the Brazilian System for the Evaluation of Psychological Tests, the EFPA model, and COTAN—found that although these systems differ in procedural and formatting details, they converge on the substantive qualities that must be reviewed (Evers, 2012). The Dutch COTAN system, for instance, rates tests on seven criteria—theoretical basis, quality of test materials, comprehensiveness of the manual, norms, reliability, construct validity, and criterion validity (Evers et al., 2010)—which map closely onto the CET-R’s four grouped dimensions (General, Validity, Reliability, and Norms). The AERA et al. (2014) Standards for Educational and Psychological Testing, likewise, organize their central concerns around validity, reliability/precision, and the fairness of scores across groups, echoing the same core evidentiary demands. This convergence across independently developed systems suggests that the gaps identified here—particularly in differential item functioning and IRT-based reliability—are unlikely to be an artifact of the specific instrument used to evaluate them, and more plausibly reflect a documentation gap that other national systems would likely detect as well.

Second, several of the highest-performing instruments in this sample are not exclusively Spanish products. The Wechsler scales, for example, have been translated and adapted in numerous countries worldwide, with the WISC-V alone available in close to a dozen adapted versions (McGill et al., 2020), and the WAIS-IV alone has separate normed adaptations for Spain, Chile, México, and Colombia (Ontiveros & Gasquoine, 2024). This international footprint cuts both ways for the interpretation of our results. On one hand, it means that at least part of the technical documentation evaluated by the CNT—theoretical foundation, internal structure—originates in, or is constrained by, decisions made at the level of the original test developer, so the strong performance of these instruments in our sample may partly reflect standards set outside Spain. On the other hand, it also means that the vulnerabilities we identified are not necessarily specific to the Spanish adaptation: methodological critiques of WISC-V translations have raised comparable concerns about equating procedures and the validation evidence accompanying adapted versions in multiple countries (McGill et al., 2020), suggesting that weaknesses such as insufficient invariance testing may recur across adaptations of the same instrument rather than being unique to the version reviewed by the CNT. Not all instruments in the sample share this international profile, however: several intelligence and aptitude batteries evaluated here—such as the BAT-7—were developed and normed directly in Spain (Arribas et al., 2013) without an equivalent international adaptation history, and for these tests our findings speak only to the Spanish context.

Taken together, these two points suggest that the present findings are informative beyond Spain in at least two ways: as evidence about specific instruments that are used, in adapted form, by practitioners in other Spanish-speaking and non-Spanish-speaking countries, and as a case study of a review system whose logic and dimensions are largely shared with the international systems that inform test-quality standards elsewhere. A direct empirical comparison between CET-R outcomes and those of COTAN or Buros reviews for the same instruments—where such overlap exists—would be a valuable next step to formally test this generalizability.

### 4.5. Study Limitations

This study has four limitations. First, the unit of analysis is the published report, not the original test manual. Reviewers already synthesize and evaluate the manual’s technical information, so this study relies on that evaluation rather than an independent reading of the manual. Second, the earliest reports were based on the original CET rather than the CET-R. Retrospective recoding into the current 14 characteristics followed previously validated criteria (Guilera & Barrios, 2025), but it may introduce some imprecision for the oldest cohorts. Third, the sample of 37 intelligence tests, while notable for this type of study, limits the statistical power of the publisher and cohort comparisons, especially for infrequently reported characteristics such as DIF analysis. Fourth, this study does not examine the validity of professional decisions made with each test, only the quality of the technical documentation available for making them. The two questions are related but not identical. Fifth, this study is fundamentally psychometric and applied in scope: it compares the technical documentation quality of tests across constructs, rather than testing or extending a theoretical model of intelligence itself. The CHC framework is used here to explain observed differences in documentation quality (Section 4.1), not as an object of empirical test. Readers interested in contributions to intelligence theory proper should not expect this study to adjudicate between competing structural models of cognitive ability.

### 4.6. Practical Implications for Test Authors and Users

Beyond the future challenges discussed in the following section, the present findings carry immediate, actionable implications for both those who develop intelligence tests and those who select and use them in professional practice.

For test users, this study offers an additional empirical criterion for choosing among instruments competing for the same assessment purpose, beyond a clinician’s tradition or familiarity with a given test. One practical caveat is worth underscoring, however: the absence of evidence on differential item functioning (DIF) and IRT-based reliability is nearly universal across the sector (Section 3.1), so this gap should not penalize any single test relative to another, but should instead prompt general caution when interpreting scores in demographically diverse populations, regardless of the instrument chosen.

For test developers (authors) and publishers, are intelligence tests currently documented well enough? The answer is nuanced: relatively well, compared with other psychological constructs (Section 3.2), but not well enough in absolute terms—even the top-performing instruments systematically omit DIF and IRT evidence. The finding of a substantial publisher effect on quality (Section 3.3), alongside the absence of a cohort effect, is itself a meaningful practical implication: it suggests that documentation quality depends on each publisher’s internal decisions, rather than on a structural limitation of the sector that the mere passage of time could resolve.

Why, then, does this gap persist even for aspects already well established by international standards? Beyond the economic argument already noted—IRT calibration and invariance studies require large, costly samples not always feasible for mid-sized publishers (Section 4.1)—a recent systematic review of psychological test revision found that existing guidelines from major international organizations focus almost exclusively on test user responsibility, offering little practical guidance to those actually carrying out a manual’s revision or update (Cronje et al., 2022). In other words, the absence of fundamental information may reflect not only a lack of economic incentive, but also a lack of a clear roadmap for those preparing test manuals.

This raises the final question: how might test developers be encouraged to adhere more closely to already well-defined standards? Evidence from other fields offers a cautionary lesson. Grant et al. (2013), in a systematic review of compliance with reporting guidelines in social and psychological intervention trials, found that the mere existence of a guideline does not ensure compliance: only 11 of 40 leading journals explicitly required adherence to a reporting guideline in their instructions to authors, and reporting standards were, on average, met in fewer than half of cases. Applied to the context of test evaluation, this suggests that voluntary recommendations alone are unlikely to be sufficient. A checklist based on the CET-R’s 14 characteristics, built into publishers’ internal manual-preparation process, would offer a concrete roadmap. Complementarily, linking CNT recognition to documentation completeness—and encouraging professional psychology associations to prioritize instruments with complete CET-R evidence in their recommendations to practitioners—could generate the kind of reputational and demand-side incentive that, according to the reporting-compliance literature, appears necessary to translate good guidelines into good practice.

### 4.7. New Challenges in Intelligence Assessment

These findings take on added relevance in light of three developments transforming cognitive assessment. The first is computerized adaptive testing. This format selects items based on the examinee’s estimated ability level, shortening the test without sacrificing precision (Weiss & Şahin, 2024). The corpus analyzed here illustrates this trend: Matrices-TAI ranks among the instruments with the highest overall psychometric quality. However, an adaptive test requires different quality evidence than a fixed-form test, such as item bank security or the continuous calibration of parameters through IRT (International Test Commission and Association of Test Publishers, 2025; Weiss & Şahin, 2024). The CET-R, originally designed for fixed-form tests, will need to incorporate specific criteria for evaluating these properties.

The second challenge is automatic item generation (AIG). These techniques make it possible to build large-scale item banks with psychometric properties known in advance, based on structural or algorithmic models of item content (von Davier, 2018). A recent critical review concludes that AIG already offers promising results for cognitive domains, although challenges remain in the empirical validation of generated items and in the transparency of the procedures used (Sommer & Arendasy, 2025). These techniques could ease shortcomings such as the limited availability of item analysis, by building that evidence into the item-construction process itself, rather than assessing it afterward.

The third challenge is the development of intelligence tests using artificial intelligence. Research on the psychometrics of artificial intelligence has begun applying classic instruments to large language models, to characterize their response profiles (Pellert et al., 2024). A recent study found high agreement between a verbal-comprehension test generated by a large language model and the WAIS-III Verbal Comprehension Index, although with a small sample and difficulties in automated scoring (Hadar-Shoval et al., 2025). This development raises a question symmetrical to the one this study addresses: if a model like the CET-R evaluates the documentation of a traditional test, what model would apply to a test whose items or scoring depend on an artificial intelligence system? Issues such as the traceability of training data or the absence of conventional manuals echo challenges previously identified in the evaluation of the quality of non-commercial tests (Abad, 2024; Guilera & Barrios, 2025).

These three developments share a common challenge. Test-review models, including the CET-R, were designed with fixed-form instruments and stable norms in mind (Evers et al., 2013; Prieto & Muñiz, 2000). Adaptive formats, algorithmically generated items, and tests based on generative artificial intelligence require rethinking which evidence should take priority. Organizations such as EFPA are already working in this direction (Hernández, 2026). However, not even intelligence tests, the domain with the strongest psychometric tradition, have yet resolved classic shortcomings such as DIF or IRT-based reliability. The immediate priority, therefore, is not only to monitor new technologies. It is to first consolidate the quality standards that already exist.

## 5. Conclusions

This study analyzed the metric quality of 37 intelligence tests evaluated by Spain’s National Test Commission across its 13 editions, compared with 78 tests of other constructs. Three main conclusions emerge.

First, intelligence tests do not provide more psychometric information than other instruments. Both groups omit around a quarter of the CET-R’s indicators, mainly for IRT-based reliability, temporal stability, and DIF.

Second, when information is reported, intelligence tests show higher quality on the General, Validity, and Norms dimensions, with moderate-to-large effect sizes. The Reliability dimension, by contrast, does not distinguish intelligence tests from other instruments. Its indicators function, instead, as a minimum standard shared across the entire sector.

Third, the publisher accounts for a substantial share of quality heterogeneity. Evaluation cohort, by contrast, does not significantly predict the quality of any characteristic once the significance level is corrected for multiple comparisons. Each publisher’s own culture matters more than the mere passage of time.

These results have immediate practical implications. Practitioners who need to choose an intelligence test can rely on this information to select instruments with the strongest, most up-to-date evidence. Publishers, in turn, now have a clear diagnosis of their priority areas for improvement: DIF analysis, temporal stability, and IRT-based reliability.

Finally, this study points to a deeper challenge for the review system itself. The CET-R—like any other framework for evaluating the quality of measurement instruments—will need to evolve to specifically assess computerized adaptive tests, automatically generated items, and tests built with generative artificial intelligence. These three developments are already present, in embryonic form, in professional and publishing practice. Consolidating classical quality standards, which remain incomplete even in the most mature psychometric domain, that of intelligence, is a necessary step. Only then will it be possible to confidently address this new generation of assessment instruments.

## Figures and Tables

**Table 1:** Missing-Data Analysis by Test Group for the CET-R’s Grouped Dimensions and Global Score.

Category	Intelligence Median [IQR]	Other Median [IQR]	Mann–Whitney *U*	*p*-Value	*p_FDR_*	Effect Size (*r*)
General	0.0% [0.0%]	0.0% [33.3%]	1249.50	0.111	0.288	0.15
Validity	25.0% [0.0%]	25.0% [50.0%]	1393.00	0.750	0.750	0.03
Reliability	50.0% [50.0%]	50.0% [0.0%]	1259.50	0.225	0.288	0.11
Norms	0.0% [0.0%]	0.0% [0.0%]	1387.50	0.234	0.288	0.11
Global Score	20.0% [23.3%]	27.5% [15.8%]	1222.00	0.177	0.288	0.12

***Note.*** IQR = interquartile range. Percentages reflect the median percentage of missing information per test within each grouped dimension (N = 37 intelligence tests vs. 78 tests of other constructs). *p_FDR_* = *p* value adjusted for the false discovery rate (Benjamini & Hochberg, 1995), computed within this family of 5 comparisons.

## Data Availability

The original data presented in the study are openly available in the reports produced by the Comisión Nacional de Test (CNT) of the Consejo General de la Psicología de España, at https://www.cop.es/test/ (accessed on 14 September 2026).

## Supplementary Materials

The following supporting information can be downloaded at https://www.mdpi.com/article/10.3390/jintelligence14090224/s1: Table S1. List of Intelligence Tests: Edition, Test Acronym and Name (English Translation in Brackets), Spanish Test Reference (English Title in Brackets), and Description of Cognitive Variables Assessed by the Test. Table S2. Welch’s *t*-Test Comparison of Psychometric Quality Scores Between Intelligence Tests and Other Constructs (With FDR-Adjusted *p* Values and Sensitivity/Power Analysis). Table S3. Kruskal–Wallis Test and Effect Sizes for the Comparison of Psychometric Quality Across Publishing Houses (With FDR-Adjusted *p* Values). Table S4. Kruskal–Wallis Test and Effect Sizes for the Comparison of Psychometric Quality Across Publication-Year Cohorts (With FDR-Adjusted *p* Values). Figure S1. Heatmap and Hierarchical Clustering Dendrogram of Psychometric Quality at the Characteristic Level.
