# Supplementary material for: Metric Quality of Intelligence Tests in Spain: Evidence from 13 National Test Commission Reviews
Source: J Intell. 2026 Sep 18;14(9):224. doi: 10.3390/jintelligence14090224 (PMC13608864; doi:10.3390/jintelligence14090224)
Supplement: Supplementary file 1 [file jintelligence-14-00224-s001.zip › Figure S1_R1.pdf]

Figure S1

Heatmap and Hierarchical Clustering Dendrogram of Psychometric Quality at the Characteristic Level

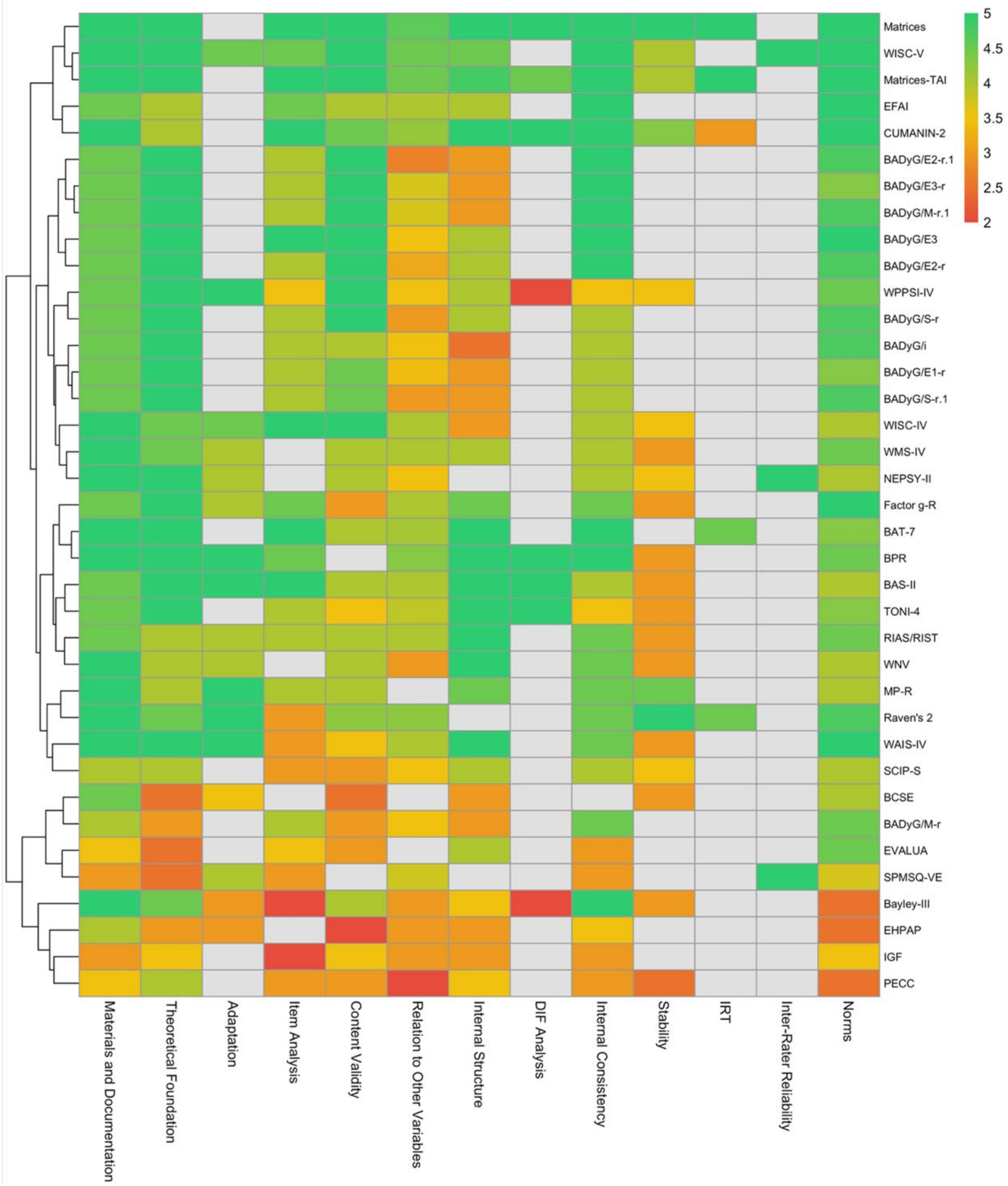

Note. Heatmap with a hierarchical clustering dendrogram (cluster analysis) showing the detailed evaluation of the 14 CET-R psychometric characteristics for each analyzed test. The color gradient ranges from green (maximum score, 5.0) to red (minimum score, 2.0); gray cells indicate unreported information or non-applicable criteria (e.g., IRT or Inter-Rater Reliability). The left-side dendrogram groups instruments based on the similarity of their psychometric quality profiles.
