# Supplementary material for: Metric Quality of Intelligence Tests in Spain: Evidence from 13 National Test Commission Reviews
Source: J Intell. 2026 Sep 18;14(9):224. doi: 10.3390/jintelligence14090224 (PMC13608864; doi:10.3390/jintelligence14090224)
Supplement: Supplementary file 1 [file jintelligence-14-00224-s001.zip › Tabla S3_R1.pdf]

Table S3. *Kruskal-Wallis Test and Effect Sizes for the Comparison of Psychometric Quality Across Publishing Houses (With FDR-Adjusted q Values)*

**Variable**	**N**	**Kruskal H**	**df**	p	p_{FDR}	η^2_H	ϵ^2
Materials & Documentation	36	15.97	3	.001	.016	.41	.46
Theoretical Foundation	36	9.99	3	.019	.037	.22	.29
Adaptation	16	3.42	2	.181	.197	.11	.23
Item Analysis	31	10.16	3	.017	.037	.27	.34
Content Validity	35	10.96	3	.012	.032	.26	.32
Relation to Other Variables	33	14.08	3	.003	.016	.38	.44
Internal Structure	34	13.90	3	.003	.016	.36	.42
DIF Analysis	8	5.33	1	.021	.037	.72	.76
Internal Consistency	35	8.04	3	.045	.060	.16	.24
Stability	21	3.37	2	.185	.197	.08	.17
IRT Analysis	5	0.14	1	.708	.708	.00	.04
Inter-Rater Reliability	2	–	–	–	–	–	–
Norms	36	4.97	3	.174	.197	.06	.14
General Score	36	9.22	3	.026	.038	.19	.26
Validity Score	36	12.54	3	.006	.019	.30	.36
Reliability Score	36	12.41	3	.006	.019	.29	.35
Norms Score	36	4.97	3	.174	.197	.06	.14
Global Score	36	9.48	3	.024	.038	.20	.27

Note. N = sample size with evaluable data for each characteristic/dimension; H = Kruskal-Wallis H statistic; df = degrees of freedom; p = level of statistical significance; η^2_H = rank-based eta-squared ($\eta^2\_H = (H - K + 1) / (N - K)$); ε^2 = epsilon-squared ($\varepsilon^2 = H / (N^2 - 1)$). The item Equivalence was excluded due to lack of data. In Inter-Rater Reliability, the sample size (N = 2) precluded running the nonparametric test. The only non-commercial intelligence test (N = 36) was excluded from this analysis. p_{FDR} = p-value adjusted for the false discovery rate (Benjamini & Hochberg, 1995), computed within this family of 16 evaluable comparisons (Inter-Rater Reliability excluded, as it was not tested).
