# Supplementary material for: Metric Quality of Intelligence Tests in Spain: Evidence from 13 National Test Commission Reviews
Source: J Intell. 2026 Sep 18;14(9):224. doi: 10.3390/jintelligence14090224 (PMC13608864; doi:10.3390/jintelligence14090224)
Supplement: Supplementary file 1 [file jintelligence-14-00224-s001.zip › Tabla S4_R1.pdf]

Table S4. *Kruskal-Wallis Test and Effect Sizes for the Comparison of Psychometric Quality Across Publication-Year Cohorts (With FDR-Adjusted p Values).*

**Variable**	**N**	**Kruskal H**	**df**	p	p_{FDR}	η_H^2	ϵ^2
Materials & Documentation	37	13.85	10	.180	.303	.15	.38
Theoretical Foundation	37	22.16	10	.014	.208	.47	.62
Adaptation	17	2.73	8	.950	.950	.00	.17
Item Analysis	32	16.62	10	.083	.208	.32	.54
Content Validity	35	16.98	10	.075	.208	.29	.50
Relation to Other Variables	34	8.57	10	.573	.661	.00	.26
Internal Structure	34	18.90	10	.042	.208	.39	.57
DIF Analysis	8	–	–	–	–	–	–
Internal Consistency	36	12.39	10	.260	.390	.10	.35
Stability	21	7.44	10	.683	.732	.00	.37
IRT Analysis	15	3.11	3	.375	.469	.11	.78
Inter-Rater Reliability	3	–	–	–	–	–	–
Norms	37	10.98	10	.359	.469	.04	.30
General Score	37	16.65	10	.082	.208	.26	.46
Validity Score	37	13.75	10	.182	.303	.15	.37
Reliability Score	37	15.89	10	.103	.221	.23	.44
Norms Score	37	10.98	10	.359	.469	.04	.30
Global Score	37	17.17	10	.071	.208	.29	.51

Note. N = sample size with evaluable data for each characteristic/dimension; H = Kruskal-Wallis H statistic; df = degrees of freedom; p = level of statistical significance; η^2_H = rank-based eta-squared (η^2_H = (H − K + 1) / (N − K)); ε^2 = epsilon-squared (ε^2 = H / (N^2 − 1)). The item Equivalence was excluded due to a low response rate. In Inter-Rater Reliability, the sample size (N = 3) prevented the execution of the nonparametric test. The DIF Analysis characteristic was omitted from the nonparametric test because the sample size (N = 8), distributed across seven temporal cohorts, did not satisfy the minimum assumptions of the Kruskal-Wallis test; p_{FDR}) = p-value adjusted for the false discovery rate (Benjamini & Hochberg, 1995), computed within this family of 15 evaluable comparisons (DIF Analysis and Inter-Rater Reliability excluded, as they were not tested). No contrast in this family survives the FDR adjustment (all $p > .05$), including Theoretical Foundation and Internal Structure, which were significant only at their uncorrected *p*-value.
