# Supplementary material for: Metric Quality of Intelligence Tests in Spain: Evidence from 13 National Test Commission Reviews
Source: J Intell. 2026 Sep 18;14(9):224. doi: 10.3390/jintelligence14090224 (PMC13608864; doi:10.3390/jintelligence14090224)
Supplement: Supplementary file 1 [file jintelligence-14-00224-s001.zip › Table S1_R1.pdf]

Table S1. *List of Intelligence Tests: Edition, Test Acronym and Name (English Translation in Brackets), Spanish Test Reference (English Title in Brackets), and Description of Cognitive Variables Assessed by the Test.*

Edition and coordinator[1,2]	Test	Test reference	Cognitive variables assessed by the test
1st Ed. (2012) J. Muñiz	WISC-IV. Escala de inteligencia de Wechsler para niños-IV [WISC-IV. Wechsler Intelligence Scale for Children-IV].	Wechsler, D. (2005). *WISC-IV: Escala de inteligencia de Wechsler para niños-IV* [WISC-IV: Wechsler Intelligence Scale for Children-IV] [Spanish adaptation by S. Corral, D. Arribas, P. Santamaría, M. J. Sueiro, & J. Pereña]. TEA Ediciones.	- Verbal Comprehension (VC): comprises 5 subtests: Similarities, Vocabulary, Comprehension, Information, and Word Reasoning.<br>- Perceptual Reasoning (PR): comprises 4 subtests: Block Design, Concepts, Matrix Reasoning, and Picture Completion.<br>- Working Memory (WM): comprises 3 subtests: Digit Span, Letter-Number Sequencing, and Arithmetic.<br>- Processing Speed (PS): comprises 3 subtests: Coding, Symbol Search, and Animal Naming.<br>- Full Scale IQ (FSIQ): composite score that summarizes overall intellectual functioning by combining the four index scores.
	EFAI. Evaluación factorial de las aptitudes intelectuales [EFAI. Factorial Assessment of Intellectual Aptitudes].	Santamaría, P., Arribas, D., Pereña, J., & Seisdedos, N. (2005). *EFAI: Evaluación factorial de las aptitudes intelectuales* [EFAI: Factorial Assessment of Intellectual Aptitudes]. TEA Ediciones.	- Spatial Aptitude (S): capacity to imagine and conceive objects and their relationships in space.<br>- Numerical Aptitude (N): capacity to reason with numerical concepts.<br>- Abstract Reasoning (R): capacity to discover the rule or logic that underlies a sequence with abstract content.<br>- Verbal Aptitude (V): capacity to reason with verbal concepts. -- Memory (M): capacity to learn and subsequently recall information.<br>- Nonverbal Intelligence (Inv): capacity to manipulate spatial relations and to reason with geometric patterns and figures; obtained by summing S and R.<br>- Verbal Intelligence (Iv): capacity to reason and solve problems that have a strong cultural component, such as verbal and numerical symbols; obtained by summing V and N.<br>- General Intelligence (Ig): global score that assesses the capacity to solve different types of problems.
	IGF. Inteligencia general y factorial [IGF. General and Factorial Intelligence].	Yuste Hernanz, C. (2001). *IGF: Inteligencia general y factorial* [IGF: General and Factorial Intelligence]. TEA Ediciones.	- Abstract Reasoning (AR): assesses reasoning processes with minimal spatial relational load.<br>- Spatial Aptitude (SpA): ability to establish relationships between figures and to manipulate them in space.<br>- Verbal Reasoning (VR): comprehension of relationships between concepts through analogies and sentence completion.<br>- Numerical Aptitude (NuA): aptitude for understanding and handling mathematical symbols in the resolution of problems.

	EVALUA. Batería psicopedagógica EVALUA [EVALUA Psychopedagogical Battery].	Martínez García, M. (Coord.), García Vidal, J., González Manjón, D., García Ortiz, B., & García Pérez, M. (2009). *Batería psicopedagógica EVALUA* [EVALUA Psychopedagogical Battery]. EOS.	- Nonverbal Intelligence (NVI): reflects fluid intelligence; obtained by summing AR and SpA.<br>- Verbal Intelligence (VI): reflects crystallized intelligence; obtained by summing VR and NuA.<br>- General Intelligence (GI): composite score that approximates the measurement of factor g; obtained by summing NVI and VI.<br>- Reasoning Foundations (Series, Classifications): assesses logical and inductive reasoning.<br>- Perceptual Organization: measures the capacity to organize and integrate stimuli.<br>- Memory-Attention: evaluates sustained attention and working memory.<br>- Analogical Thinking: assesses the capacity for analogical reasoning by establishing relationships between concepts.<br>- Calculation and Problem Solving: evaluates basic mathematical skills and the capacity to solve numerical problems.<br>- Reflexivity: measures the degree of control over cognitive impulsivity.<br>- Other constructs such as Adaptation and the psychosocial climate scale.
2nd Ed. (2013) V. Ponsoda	BAS-II. Escalas de aptitudes intelectuales [BAS-II. Scales of Intellectual Ability].	Elliott, C. D. (2011). *BAS-II: Escalas de aptitudes intelectuales* [BAS-II: Scales of Intellectual Ability] [Spanish adaptation by D. Arribas & S. Corral]. TEA Ediciones.	- General Index (GI): estimate of general ability combining verbal and nonverbal components.<br>- General Nonverbal Index (GNVI): equivalent to GI but minimizing the verbal component, useful in the presence of language difficulties.<br>- Verbal Index (VI): measures the acquisition of verbal concepts and vocabulary.<br>- Perceptual Reasoning (PR; up to 5 years 11 months).<br>- Nonverbal Reasoning (NVR; from 6 years 0 months).<br>- Spatial Index (SI).
	RIAS/RIST. Escalas de inteligencia de Reynolds y Test de inteligencia breve de Reynolds [RIAS/RIST. Reynolds Intellectual Assessment Scales and Reynolds Intellectual Screening Test].	Reynolds, C. R., & Kamphaus, R. W. (2009). *RIAS: Escalas de inteligencia de Reynolds y RIST: Test de inteligencia breve de Reynolds* [RIAS: Reynolds Intellectual Assessment Scales and RIST: Reynolds Intellectual Screening Test] [Spanish adaptation by P. Santamaría & I. Fernández]. TEA Ediciones.	- Guess What.<br>- Verbal Analogies.<br>- Incomplete Figures.<br>- Categories.<br>- Verbal Memory.<br>- Nonverbal Memory.<br>- Verbal Intelligence Index (VI): combines Guess What and Verbal Analogies; reflects crystallized intelligence.<br>- Nonverbal Intelligence Index (NVI): combines Incomplete Figures and Categories; reflects fluid intelligence.<br>- General Memory Index (GMI): combines Verbal Memory and Nonverbal Memory, providing a global measure of memory.<br>- General Intelligence Index (GI): composite score derived from the four intelligence subtests (verbal and nonverbal), representing factor g.

			- RIST Index: brief screening score (based on Guess What and Categories) that estimates the risk of intellectual impairment.
	WNV. Escala no verbal de aptitud intelectual de Wechsler [WNV. Wechsler Nonverbal Scale of Ability].	Wechsler, D., & Naglieri, J. A. (2011). *WNV: Escala no verbal de aptitud intelectual de Wechsler* [WNV: Wechsler Nonverbal Scale of Ability] [Spanish adaptation by F. Vallar, E. de la Guía, & E. Paradell]. Pearson Educación.	The WNV (from 5 to 21 years 11 months) is composed of nonverbal subtests and is useful for individuals with language barriers.<br>- General Intellectual Ability (factor g): in confirmatory factor analysis, the four subtests load on a single general intelligence factor that adequately describes the structure of the test. It has an abbreviated form composed of two subtests.
	MP-R. Escalas de desarrollo Merrill-Palmer revisadas [MP-R. Merrill-Palmer–Revised Developmental Scales].	Roid, G. H., Sampers, J. L., Anderson, G., Erickson, J., & Post, P. (2011). *MP-R: Escalas de desarrollo Merrill-Palmer revisadas* [MP-R: Merrill-Palmer–Revised Developmental Scales] [Spanish adaptation by F. Sánchez-Sánchez, P. Santamaría, I. Fernández, & D. Arribas]. TEA Ediciones.	- Global Index (GI): general measure of cognitive development according to the Cattell-Horn-Carroll model.<br>- Cognition (C): assesses verbal and nonverbal reasoning.<br>- Memory (M): measures short-term memory.<br>- Processing Speed (P): evaluates the speed of mental information processing.<br>- Visomotor Coordination (VMC): measures integration between perception and motor response.<br>- Fine Motor (FM).<br>- Gross Motor (GM).<br>- Receptive Language (RL).<br>- Other constructs: socioemotional development, adaptive behavior and self-care, and temperament style.
3rd Ed. (2014) A. Hernández	WAIS-IV. Escala de inteligencia de Wechsler para adultos-IV [WAIS-IV. Wechsler Adult Intelligence Scale-IV].	Wechsler, D. (2012). *WAIS-IV: Escala de inteligencia de Wechsler para adultos-IV* [WAIS-IV: Wechsler Adult Intelligence Scale-IV] [Spanish adaptation by E. de la Guía, A. Hernández, E. Paradell, & F. Vallar]. Pearson Educación.	- Verbal Comprehension Index (VCI). Subtests: Similarities, Vocabulary, Information, and Comprehension.<br>- Perceptual Reasoning Index (PRI). Subtests: Block Design, Matrix Reasoning, Visual Puzzles, Figure Weights, and Picture Completion.<br>- Working Memory Index (WMI). Subtests: Digit Span, Arithmetic, and Letter-Number Sequencing.<br>- Processing Speed Index (PSI). Subtests: Symbol Search, Coding, and Cancellation.<br>- Full Scale IQ (FSIQ): composite score representing general intellectual ability; combines information from the four cognitive indices.
	BCSE. Test breve para la evaluación del estado cognitivo [BCSE. Brief Cognitive Status Exam].	Wechsler, D. (2013). *BCSE: Test breve para la evaluación del estado cognitivo* [BCSE: Brief Cognitive Status Exam]. Pearson Educación.	- Orientation.<br>- Time estimation.<br>- Naming.<br>- Mental control.<br>- Clock drawing.<br>- Incidental recall (of the stimuli previously presented in the Naming task).<br>- Inhibition.<br>- Verbal production.

	BADyG/M-r. Batería de Aptitudes Diferenciales y Generales, Nivel M renovado [BADyG/M-r. Differential and General Aptitudes Battery, Level M, renewed].	Yuste Hernanz, C., Yuste Peña, D., Martínez Arias, R., & Galve Manzano, J. L. (2014). *BADyG/M-r: Batería de Aptitudes Diferenciales y Generales, Nivel M renovado* [BADyG/M-r: Differential and General Aptitudes Battery, Level M, renewed]. CEPE, S.L.	Designed for students in 1st, 2nd, 3rd, and 4th year of ESO (12–16 years). It allows the assessment of:<br>- Logical Reasoning: global estimate obtained from the three reasoning tests (Rv, Rn, Re).<br>- Verbal Factor: global estimate obtained from two tests (Rv and Vv).<br>- Numerical Factor: global estimate obtained from two tests (Rn and Nn).<br>- Visuospatial Factor: global estimate obtained from two tests (Re and Ge).<br>- General Intelligence (GI): global construct evaluated through the six basic tests and their derived factors.
4th Ed. (2015) P. Elosua	WPPSI-IV. Escala de inteligencia de Wechsler para preescolar y primaria-IV [WPPSI-IV. Wechsler Preschool and Primary Scale of Intelligence-IV].	Wechsler, D. (2014). *WPPSI-IV: Escala de inteligencia de Wechsler para preescolar y primaria-IV* [WPPSI-IV: Wechsler Preschool and Primary Scale of Intelligence-IV] [Spanish adaptation by the Pearson Clinical R&D Department]. Pearson Educación.	The WPPSI-IV assesses children from 2 years 6 months to 7 years 7 months and provides different indices depending on age range.<br>- Full Scale IQ (FSIQ): general score of intellectual ability obtained from combinations of subtest scores.<br>- Verbal Comprehension.<br>- Visuospatial Ability.<br>- Working Memory.<br>- Fluid Reasoning.<br>- Processing Speed.<br>- Vocabulary Acquisition.<br>- Nonverbal Ability: reflects overall intellectual functioning based on subtests without a verbal component.<br>- General Ability: estimate of intellectual aptitude based on a subset of subtests, with its own theoretical basis.<br>- Cognitive Proficiency: reflects other aspects of cognitive aptitude, emphasizing working memory and processing speed.
	SCIP-S. Screening del deterioro cognitivo en psiquiatría [SCIP-S. Screening for Cognitive Impairment in Psychiatry].	Pino, O., Guilera, G., Rojo, J. E., Gómez-Benito, J., & Purdon, S. E. (2014). *SCIP-S: Screening del deterioro cognitivo en psiquiatría* [SCIP-S: Screening for Cognitive Impairment in Psychiatry]. TEA Ediciones.	- Immediate Verbal Learning (IVL): assesses the capacity to learn a word list across three consecutive trials.<br>- Working Memory (WM): measures retention of consonant trigrams under distraction conditions.<br>- Verbal Fluency (VF): assesses the capacity to generate words beginning with a given letter in 30 seconds, across two trials.<br>- Delayed Verbal Learning (DVL): measures delayed recall of the word list initially learned.<br>- Processing Speed (PS): evaluates processing speed through translation of letters into a Morse-type code within a limited time.<br>- General Index: global score combining performance across the five subtests.
	WMS-IV. Escala de memoria de Wechsler-	Wechsler, D. (2013). *WMS-IV: Escala de memoria de Wechsler-IV* [WMS-IV: Wechsler Memory Scale-IV] [Spanish	Designed for individuals aged 16 to 89 years and composed of two batteries: the Adult Battery (16-69 years) and the Older Adult Battery (65-89 years). It provides five memory indices:

	IV [WMS-IV. Wechsler Memory Scale-IV].	adaptation by the Pearson Clinical & Talent Assessment R&D Department]. Pearson Educación.	- Auditory Memory Index (AMI).<br>- Visual Memory Index (VMI).<br>- Immediate Memory Index (IMI).<br>- Delayed Memory Index (DMI).<br>- Visual Working Memory Index (VWMI).
	PECC. Prueba para la Evaluación de la Cognición Cotidiana [PECC. Everyday Cognition Assessment Test].	Menor de Gaspar Pinilla, J., & Labra Pérez, J. A. (2014). *PECC: Prueba para la Evaluación de la Cognición Cotidiana* [PECC: Everyday Cognition Assessment Test]. EOS.	- Use of Medication and Health Care Management: assesses the capacity to manage medications and carry out health-related procedures.<br>- Administrative Tasks: measures the capacity to perform common bureaucratic procedures.<br>- Financial Management: evaluates handling of money and everyday financial matters.<br>- Meal Preparation: assesses the planning capacity required to prepare a recipe.<br>- Use of Transport: evaluates orientation and the capacity to use means of transport.<br>- Shopping: measures calculation and decision making involved in purchasing.<br>- Total Score (impairment in IADL): global indicator that synthesizes cognitive performance in everyday tasks.
	EHPAP. Evaluación de habilidades y potencial de aprendizaje en preescolares [EHPAP. Assessment of Skills and Learning Potential in Preschoolers].	Calero, M. D., Robles, M., Márquez, J., & De la Osa, P. (2009). *EHPAP: Evaluación de habilidades y potencial de aprendizaje en preescolares* [EHPAP: Assessment of Skills and Learning Potential in Preschoolers]. EOS.	- Classification: assesses the capacity to group according to different criteria.<br>- Auditory Memory: measures retention of orally presented information.<br>- Visual Memory: measures retention of visually presented information.<br>- Series: measures sequential reasoning through completion of patterns.<br>- Verbal Planning: measures the capacity to organize strategies verbally.<br>- Other constructs: Perspective Taking, and a Behavioral Observation Scale (BOS) to assess self-regulation, persistence, tolerance to frustration, flexibility, motivation, interactivity, and receptivity.
5th Ed. (2016) E. Fonseca	BADyG/E3. Batería de Aptitudes Diferenciales y Generales, Nivel E3 renovado [BADyG/E3. Differential and General Aptitudes Battery, renewed Level E-3].	Yuste Hernanz, C. (2011). *BADyG-E3: Batería de Aptitudes Diferenciales y Generales renovado E-3* [BADyG-E3: Differential and General Aptitudes Battery, renewed Level E-3]. CEPE, S.L.	Designed for students in 5th and 6th year of Primary Education (10–12 years). It allows the assessment of:<br>- Logical Reasoning: global estimate obtained from the three reasoning tests (Rv, Rn, Re).<br>- Verbal Factor: global estimate obtained from two tests (Rv and Vv).<br>- Numerical Factor: global estimate obtained from two tests (Rn and Nn).<br>- Visuospatial Factor: global estimate obtained from two tests (Re and Ge).<br>- General Intelligence (GI): global construct evaluated through the six basic tests and their derived factors.
	WISC-V. Escala de inteligencia de Wechsler para niños-V [WISC-V. Wechsler	Wechsler, D. (2015). *WISC-V: Escala de inteligencia de Wechsler para niños-V* [WISC-V: Wechsler Intelligence Scale for Children-V] [Spanish adaptation by A.	Consists of 15 subtests organized in three levels: the full scale (Full Scale IQ), primary indices, and secondary indices.<br>- Full Scale IQ: obtained from seven subtests (Similarities, Vocabulary, Block Design, Matrix Reasoning, Figure Weights, Digit Span, and Coding).<br>- Verbal Comprehension Index (VCI): Similarities and Vocabulary.

	Intelligence Scale for Children-V].	Hernández, C. Aguilar, E. Paradell, & F. Vallar]. Pearson Educación.	- Visuospatial Index (VSI): Block Design and Visual Puzzles. Fluid Reasoning Index (FRI): Matrix Reasoning and Figure Weights.<br>- Working Memory Index (WMI): Digit Span and Picture Span.<br>- Processing Speed Index (PSI): Coding and Symbol Search.<br>- Quantitative Reasoning Index (QRI): Figure Weights and Arithmetic.<br>- Auditory Working Memory Index (AWMI): Digit Span and Letter-Number Sequencing.
	NEPSY-II. Batería neuropsicológica infantil (2.ª ed.) [NEPSY-II. Developmental Neuropsychological Assessment, 2nd edition].	Korkman, M., Kirk, U., & Kemp, S. (2014). *NEPSY-II: Batería neuropsicológica infantil (2.ª ed.)* [NEPSY-II: Developmental Neuropsychological Assessment, 2nd edition] [Spanish adaptation by Centro de Rehabilitación Neurológica-FIVAN and the University of Seville, with the Pearson Clinical & Talent Assessment R&D Department]. Pearson Educación.	- Attention and Executive Function: assessed through subtests such as Auditory Attention and Response Set, Animal Sorting, Design Fluency, Inhibition, and Clocks.<br>- Language: assessed through subtests such as Comprehension of Instructions, Body Part Naming and Identification, Verbal Fluency, and Speeded Naming.<br>- Memory and Learning: assessed through subtests such as Word List Interference, Memory for Faces, Memory for Designs, List Memory, Names Memory, Narrative Memory, and Sentence Repetition (some with delayed versions).<br>- Visuospatial Processing: assessed through Block Construction, Design Copying, Route Finding, Arrows, Picture Puzzles, and Geometric Puzzles.
6th Ed. (2017)<br>M. D. Hidalgo	BADyG/E2-r. Batería de Aptitudes Diferenciales y Generales, Nivel E2 renovado [BADyG/E2-r. Differential and General Aptitudes Battery, Level E2, renewed].	Yuste Hernanz, C., & Yuste Peña, D. (2011). *BADyG/E2-r: Batería de Aptitudes Diferenciales y Generales, Nivel E2 renovado* [BADyG/E2-r: Differential and General Aptitudes Battery, Level E2, renewed]. CEPE, S.L.	Designed for students in 3rd and 4th year of Primary Education (8–9 years). It allows the assessment of:<br>- Logical Reasoning: global estimate obtained from the three reasoning tests (Rv, Rn, Re).<br>- Verbal Factor: global estimate obtained from two tests (Rv and Vv).<br>- Numerical Factor: global estimate obtained from two tests (Rn and Nn).<br>- Visuospatial Factor: global estimate obtained from two tests (Re and Ge).<br>- General Intelligence (GI): global construct evaluated through the six basic tests and their derived factors.
	BADyG/S-r. Batería de Aptitudes Diferenciales y Generales, Nivel S renovado [BADyG/S-r. Differential and General Aptitudes Battery, Level S, renewed].	Yuste Hernanz, C., Yuste Peña, D., Martínez Arias, R., & Galve Manzano, J. L. (2011). *BADyG/S-r: Batería de Aptitudes Diferenciales y Generales, Nivel S renovado* [BADyG/S-r: Differential and General Aptitudes Battery, Level S, renewed]. CEPE, S.L.	Designed for students in the final two years of upper secondary education (Spanish Bachillerato) (16–20 years). It allows the assessment of:<br>- Logical Reasoning: global estimate obtained from the three reasoning tests (Rv, Rn, Re).<br>- Verbal Factor: global estimate obtained from two tests (Rv and Vv).<br>- Numerical Factor: global estimate obtained from two tests (Rn and Nn).<br>- Visuospatial Factor: global estimate obtained from two tests (Re and Ge).<br>- General Intelligence (GI): global construct evaluated through the six basic tests and their derived factors.
	BAT-7. Batería de Aptitudes de TEA [BAT-	Arribas, D., Santamaría, P., Sánchez-Sánchez, F., & Fernández-Pinto, I. (2015).	- Verbal Aptitude (V).<br>- Spatial Aptitude (S).<br>- Attention (A).

	7. TEA Aptitude Battery].	*BAT-7: Batería de Aptitudes de TEA* [BAT-7: TEA Aptitude Battery]. TEA Ediciones.	- Concentration (CON).<br>- Reasoning (R).<br>- Numerical Aptitude (N).<br>- Mechanical Aptitude (M).<br>- Spelling (O).<br>- Fluid Intelligence (Gf): composed of R and N.<br>- Crystallized Intelligence (Gc): composed of V and O.<br>- Attentional Style: score derived from A and CON.<br>- General Ability (g): index in which all aptitudes take part; indicates a superior and general capacity to understand situations, reason, solve complex problems, and act effectively when faced with difficult or novel tasks.
	BPR. Batería de Pruebas de Razonamiento [BPR. Reasoning Test Battery].	Elosua, P., & Almeida, L. S. (2016). *BPR: Batería de Pruebas de Razonamiento* [BPR: Reasoning Test Battery]. TEA Ediciones.	- Abstract Reasoning (Ra).<br>- Verbal Reasoning (Rv).<br>- Numerical Reasoning (Rn).<br>- Practical Reasoning (Rp).<br>- Spatial Reasoning (Re).<br>- Mechanical Reasoning (Rm).<br>- General Reasoning (Rg): global index that reflects the level on the general reasoning factor; obtained by combining all tests.
	Matrices. Test de Inteligencia General [Matrices. General Intelligence Test].	Sánchez-Sánchez, F., Santamaría, P., & Abad, F. J. (2015). *Matrices: Test de Inteligencia General* [Matrices: General Intelligence Test]. TEA Ediciones.	General Intelligence: estimated through a nonverbal abstract reasoning task based on graphic matrices, grounded in the Cattell–Horn–Carroll (CHC) model.
7th Ed. (2018) L. Gómez	Factor g-R. Test de Factor g (Revisado) [Factor g-R. g Factor Test (Revised)].	Cattell, R. B., Cattell, A. K., & Weiss, R. H. (2017). *Factor "g"-R: Test de Factor "g" (Revisado)* [Factor "g"-R: g Factor Test (Revised)] [Spanish adaptation by the TEA Ediciones R&D+i Department]. TEA Ediciones.	- Series: assesses the capacity to complete incomplete and progressive series of figures.<br>- Classification: the task is to identify, among a group of figures, which one is different from the others according to a logical criterion.<br>- Matrices: involves completing a matrix of drawings based on abstract relationships among its elements.<br>- Conditions: the task is to choose the alternative that meets the same conditions as a reference figure.<br>- Factor g / Fluid Intelligence: general index that indicates the capacity to understand and relate complex ideas, work efficiently with abstract content, and draw logical conclusions through deductive and inductive processes; it estimates fluid intelligence (Gf) while reducing the influence of cultural factors or verbal fluency.
8th Ed. (2019) C. Viladrich	BADyG/i. Batería de Aptitudes Diferenciales y Generales, Nivel	Yuste Hernanz, C., & Yuste Peña, D. (2019). *BADyG/i: Batería de Aptitudes Diferenciales y Generales, Nivel Infantil* [BADyG/i:	Designed for children aged 4 to 6 years (second cycle of Early Childhood Education). It allows the assessment of:

Infantil [BADyG/i. Differential and General Aptitudes Battery, Preschool Level].	Differential and General Aptitudes Battery, Preschool Level]. CEPE, S.L.	- General Verbal Intelligence (GVI): child's capacity to understand and reason with verbal concepts; obtained by combining the tests Quantitative-Numerical Concepts, Information, and Graphic Vocabulary.<br>- General Nonverbal Intelligence (GNVI): capacity to understand and reason primarily using geometric figures or drawings; obtained by combining Nonverbal Mental Ability, Reasoning with Figures, and Puzzles.<br>- General Intelligence (GI): child's general capacity to establish relationships among abstract concepts using varied mental contents; obtained by summing GVI and GNVI.
BADyG/E1-r. Batería de Aptitudes Diferenciales y Generales, Nivel E1 renovado [BADyG/E1-r. Differential and General Aptitudes Battery, Level E1, renewed].	Yuste Hernanz, C., & Yuste Peña, D. (2019). *BADyG/E1-r: Batería de Aptitudes Diferenciales y Generales, Nivel E1 renovado* [BADyG/E1-r: Differential and General Aptitudes Battery, Level E1, renewed]. CEPE, S.L.	Designed for students in 1st and 2nd year of Primary Education (6-8 years). It allows the assessment of:<br>- Logical Reasoning: global estimate obtained from the three reasoning tests (Rv, Rn, Re).<br>- Verbal Factor: global estimate obtained from two tests (Rv and Vv).<br>- Numerical Factor: global estimate obtained from two tests (Rn and Nn).<br>- Visuospatial Factor: global estimate obtained from two tests (Re and Ge).<br>- General Intelligence (GI): global construct evaluated through the six basic tests and their derived factors.
BADyG/E2-r. Batería de Aptitudes Diferenciales y Generales, Nivel E2 renovado [BADyG/E2-r. Differential and General Aptitudes Battery, Level E2, renewed].	Yuste Hernanz, C., & Yuste Peña, D. (2019). *BADyG/E2-r: Batería de Aptitudes Diferenciales y Generales, Nivel E2 renovado* [BADyG/E2-r: Differential and General Aptitudes Battery, Level E2, renewed]. CEPE, S.L.	Designed for students in 3rd and 4th year of Primary Education (8-9 years). It allows the assessment of:<br>- Logical Reasoning: global estimate obtained from the three reasoning tests (Rv, Rn, Re).<br>- Verbal Factor: global estimate obtained from two tests (Rv and Vv).<br>- Numerical Factor: global estimate obtained from two tests (Rn and Nn).<br>- Visuospatial Factor: global estimate obtained from two tests (Re and Ge).<br>- General Intelligence (GI): global construct evaluated through the six basic tests and their derived factors.
BADyG/E3-r. Batería de Aptitudes Diferenciales y Generales, Nivel E3 renovado [BADyG/E3-r. Differential and General Aptitudes Battery, Level E3, renewed].	Yuste Hernanz, C., Yuste Peña, D., Martínez Arias, R., & Galve Manzano, J. L. (2019). *BADyG/E3-r: Batería de Aptitudes Diferenciales y Generales, Nivel E3 renovado* [BADyG/E3-r: Differential and General Aptitudes Battery, Level E3, renewed]. CEPE, S.L.	Designed for students in 5th and 6th year of Primary Education (10-12 years). It allows the assessment of:<br>- Logical Reasoning: global estimate obtained from the three reasoning tests (Rv, Rn, Re).<br>- Verbal Factor: global estimate obtained from two tests (Rv and Vv).<br>- Numerical Factor: global estimate obtained from two tests (Rn and Nn).<br>- Visuospatial Factor: global estimate obtained from two tests (Re and Ge).<br>- General Intelligence (GI): global construct evaluated through the six basic tests and their derived factors.
BADyG/M-r. Batería de Aptitudes Diferenciales y Generales, Nivel M	Yuste Hernanz, C., Yuste Peña, D., Martínez Arias, R., & Galve Manzano, J. L. (2019). *BADyG/M-r: Batería de Aptitudes*	Designed for students in 1st, 2nd, 3rd, and 4th year of ESO (12-16 years). It allows the assessment of:

	renovado [BADyG/M-r. Differential and General Aptitudes Battery, Level M, renewed].	*Diferenciales y Generales, Nivel M renovado* [BADyG/M-r: Differential and General Aptitudes Battery, Level M, renewed]. CEPE, S.L.	- Logical Reasoning: global estimate obtained from the three reasoning tests (Rv, Rn, Re).<br>- Verbal Factor: global estimate obtained from two tests (Rv and Vv).<br>- Numerical Factor: global estimate obtained from two tests (Rn and Nn).<br>- Visuospatial Factor: global estimate obtained from two tests (Re and Ge).<br>- General Intelligence (GI): global construct evaluated through the six basic tests and their derived factors.
	BADyG/S-r. Batería de Aptitudes Diferenciales y Generales, Nivel S renovado [BADyG/S-r. Differential and General Aptitudes Battery, Level S, renewed].	Yuste Hernanz, C., Yuste Peña, D., Martínez Arias, R., & Galve Manzano, J. L. (2019). *BADyG/S-r: Batería de Aptitudes Diferenciales y Generales, Nivel S renovado* [BADyG/S-r: Differential and General Aptitudes Battery, Level S, renewed]. CEPE, S.L.	Designed for students in the final two years of upper secondary education (Spanish Bachillerato; 16-20 years). It allows the assessment of:<br>- Logical Reasoning: global estimate obtained from the three reasoning tests (Rv, Rn, Re).<br>- Verbal Factor: global estimate obtained from two tests (Rv and Vv).<br>- Numerical Factor: global estimate obtained from two tests (Rn and Nn).<br>- Visuospatial Factor: global estimate obtained from two tests (Re and Ge).<br>- General Intelligence (GI): global construct evaluated through the six basic tests and their derived factors.
	TONI-4. Test de Inteligencia No Verbal [TONI-4. Test of Nonverbal Intelligence].	Brown, L., Sherbenou, R. J., & Johnsen, S. K. (2019). *TONI-4: Test de Inteligencia No Verbal* [TONI-4: Test of Nonverbal Intelligence] [Spanish adaptation by B. Ruiz-Fernández]. TEA Ediciones.	- General Intelligence: the TONI-4 consists of a single scale that assesses global intelligence through abstract reasoning and problem solving. The aim of the test was to obtain a good measure of the theoretical construct g. Its nonverbal format eliminates the influence of language, making it suitable for individuals with verbal, auditory, or motor difficulties.
9th Ed. (2020) L. Lozano	Raven's 2. Matrices progresivas de Raven-2 [Raven's 2. Raven's 2 Progressive Matrices].	Pearson Clinical & Talent Assessment. (2019). *Raven's 2: Matrices progresivas de Raven-2* [Raven's 2: Raven's 2 Progressive Matrices] [Spanish adaptation by A. Hernández, C. Aguilar, E. Paradell, & F. Vallar; Pearson Clinical & Talent Assessment R&D Department]. Pearson Educación.	- General Cognitive Functioning / Factor g: as one of the fundamental components of intelligence (factor g), the test provides a rapid estimate of the subject's general cognitive functioning, calculated using the Rasch model (IRT).
	Bayley-III. Escalas Bayley de desarrollo infantil-III [Bayley-III. Bayley Scales of Infant Development-III].	Bayley, N. (2015). *Bayley-III: Escalas Bayley de desarrollo infantil-III* [Bayley-III: Bayley Scales of Infant Development-III] [Spanish adaptation by CDIAP Parc Taulí, the University of Murcia, and the Pearson Clinical & Talent Assessment R&D Department]. Pearson Educación.	- Cognitive Scale: evaluates sensorimotor development, exploration and manipulation of objects, concept formation, memory, attention, and visual preference.<br>- Receptive Communication Scale.<br>- Expressive Communication Scale.<br>- Fine Motor Scale.<br>- Gross Motor Scale.
11th Ed. (2022) G. Guillera & M. Barrios	CUMANIN-2. Cuestionario de Madurez	Portellano, J. A., Mateos, R., Martínez-Arias, R., & Sánchez-Sánchez, F. (2021). *CUMANIN-2: Cuestionario de Madurez*	- Sensorimotor Development Index (SDI): composite index formed by the subscales Psychomotricity, Spatial, Visuoperception, Rhythm, and Attention.

	Neuropsicológica Infantil [CUMANIN-2. Children's Neuropsychological Maturity Questionnaire].	*Neuropsicológica Infantil* [CUMANIN-2: Children's Neuropsychological Maturity Questionnaire]. Hogrefe TEA Ediciones.	- Memory and Learning Index (MLI): composite index formed by the subscales Visual Memory and Verbal Memory.<br>- Language Development Index (LDI): composite index formed by Articulatory Language, Expressive Language, Receptive Language, and Verbal Fluency.<br>- Neuropsychological Development Index (NDI): global score derived from the three previous indices.
12th Ed. (2023) P. Recio-Saboya	Matrices-TAI. Test Adaptativo de Inteligencia General [Matrices-TAI. Adaptive General Intelligence Test].	Abad, F. J., Sánchez-Sánchez, F., & Santamaría, P. (2020). *Matrices-TAI: Test Adaptativo de Inteligencia General* [Matrices-TAI: Adaptive General Intelligence Test]. TEA Ediciones.	- General Intelligence: estimated through an abstract reasoning task based on graphic matrices, administered in a computerized adaptive test (CAT) format.
12th Ed. (2023) P. Recio-Saboya	SPMSQ-VE. Cuestionario de Pfeiffer para la detección del deterioro cognitivo (versión española) [SPMSQ-VE. Pfeiffer Questionnaire for Detecting Cognitive Impairment (Spanish version)].	Martínez de la Iglesia, J., Dueñas, R., Onís, M. C., Aguado, C., Colomer, C., & Luque, R. (2001). Adaptación y validación al castellano del cuestionario de Pfeiffer (SPMSQ) para detectar la existencia de deterioro cognitivo en personas mayores de 65 años [Adaptation and validation of the Pfeiffer questionnaire (SPMSQ) into Spanish to detect cognitive impairment in people over 65 years]. *Medicina Clínica, 117*(4), 129–134. [Adaptation of E. Pfeiffer's Short Portable Mental Status Questionnaire, 1975].	- Orientation.<br>- Short-term memory.<br>- Long-term memory.<br>- Calculation / Concentration ability.

Note. (1) The WAIS-III was evaluated in the 1st edition. However, its report does not include published scores for any dimension due to a dispute over publishing rights. For this reason, it was excluded from all the analysis, leaving the final N at 37. (2) In the 10th and 13th editions, no intelligence tests were identified.
