# Supplementary material for: Metric Quality of Intelligence Tests in Spain: Evidence from 13 National Test Commission Reviews
Source: J Intell. 2026 Sep 18;14(9):224. doi: 10.3390/jintelligence14090224 (PMC13608864; doi:10.3390/jintelligence14090224)
Supplement: Supplementary file 1 [file jintelligence-14-00224-s001.zip › Table S2_R1.pdf]

Table S2. *Welch's t-Test Comparison of Psychometric Quality Scores Between Intelligence Tests and Other Constructs (With FDR-Adjusted p Values and Sensitivity/Power Analysis)*

Variable	Intelligence Tests			Other Constructs			Welch's t	df	*p*	p_{FDR}	Hedges' *g*	Observed Power	MDE (*g*, 80% power)
	n	*M*	*SD*	*n*	*M*	*SD*							
Materials & Documentation	37	4.51	0.55	58	4.18	0.74	2.54	90.81	.013	.028	0.51	0.67	0.60
Theoretical Foundation	37	4.41	0.82	66	3.96	0.87	2.58	78.82	.012	.028	0.52	0.71	0.58
Adaptation	17	4.26	0.69	32	4.03	0.92	1.00	41.65	.323	.392	0.28	0.15	0.86
Item Analysis	32	3.97	0.83	50	3.67	0.77	1.63	62.63	.108	.184	0.37	0.37	0.64
Content Validity	35	4.08	0.83	58	3.56	0.84	2.89	72.51	.005	.014	0.61	0.81	0.61
Relation to Other Variables	34	3.64	0.58	44	3.43	0.85	1.31	74.97	.194	.275	0.29	0.24	0.65
Internal Structure	34	3.96	0.84	54	3.31	1.24	2.98	85.51	.004	.014	0.62	0.80	0.62
DIF Analysis	8	4.19	1.36	19	3.66	1.18	0.96	11.67	.357	.405	0.39	0.14	1.23
Internal Consistency	36	4.31	0.68	60	4.09	0.72	1.45	77.45	.152	.235	0.30	0.29	0.60
Stability	21	3.49	0.71	32	3.37	0.77	0.59	45.28	.559	.594	0.16	0.09	0.80
IRT Analysis	5	4.40	0.82	5	4.20	0.45	0.48	6.18	.649	.649	0.26	0.07	2.02
Inter-Rater Reliability	3	5.00	0.00	6	4.00	0.89	2.74	5.00	.041	.077	1.33	0.37	2.31
Norms	37	4.32	0.68	63	3.85	0.63	3.43	71.27	.001	.006	0.71	0.92	0.59
General Score	37	4.30	0.58	74	3.92	0.66	3.12	81.46	.003	.013	0.61	0.85	0.57
Validity Score	37	3.88	0.57	73	3.44	0.76	3.40	92.35	.001	.006	0.65	0.89	0.57
Reliability Score	37	4.07	0.66	68	3.92	0.69	1.07	77.18	.288	.377	0.21	0.17	0.58
Norms Score	37	4.32	0.68	63	3.85	0.63	3.43	71.27	.001	.006	0.71	0.92	0.59
Global Score	37	4.11	0.51	78	3.75	0.58	3.35	80.23	.001	.006	0.65	0.90	0.56

***Note*.** All items are rated on a 1–5 scale. Sample sizes (n) vary across features due to available-case analysis (excluding missing/non-applicable values). df values reflect Satterthwaite's approximation for Welch's t-test. The p_{FDR} column corresponds to the p-value corrected using the Benjamini-Hochberg procedure to control the false discovery rate (FDR), considering the m = 17 comparisons in this table. Hedges' g uses unpooled standard deviations. *Observed Power* is the post hoc power to detect the observed effect size (g) given the actual sample sizes, at α = .05, two-tailed. *MDE (g, 80% power)* is the minimum effect size (Hedges' g) that this comparison's sample

sizes could detect with 80% power at $\alpha = .05$, two-tailed; comparisons whose observed g falls below this value should be interpreted with caution, as the design lacks adequate power to rule out effects of that magnitude.
